# Enhancing Tumor Perfusion and Nanomedicine Delivery by Modulating Vascular Tone with Methyl Palmitate Nanoparticles

**DOI:** 10.1021/acsnano.6c03988

**Published:** 2026-07-17

**Authors:** Roberto Palomba, Elizabeth Isaac, Raffaele Spanò, Federica Piccardi, Benedict McLarney, Elana Apfelbaum, Nermin Mostafa, Hsiao-Ting Hsu, Jan Grimm, Paolo Decuzzi

**Affiliations:** † 121451 Laboratory of Nanotechnology for Precision Medicine − Fondazione Istituto Italiano di Tecnologia, Via Morego 30, Genova 16163, Italy; ‡ Memorial Sloan Kettering Cancer Center, Sloan Kettering Institute (SKI), 5803 Molecular Pharmacology Program, 1275 York Avenue, New York City, New York Z2025, United States; § School of Medicine/Division of Oncology, Center for Clinical Sciences Research, Stanford University, 269 Campus Drive, Stanford, California 94305, United States

**Keywords:** tumor perfusion, vascular permeability, nitric oxide, methyl palmitate, nanomedicine

## Abstract

Despite a few clinical successes, the efficacy of cancer nanomedicines remains limited by rapid clearance by the mononuclear phagocytic system and poor permeation across the abnormal tumor vasculature. We previously showed that methyl palmitate nanoparticles (MPN) can safely and reversibly inhibit the phagocytic activity of immune cells for several hours, thereby improving tumor accumulation and the efficacy of systemically administered nanomedicines. Here, we demonstrate that, on a shorter time scale, MPN can induce vasodilation, introducing an additional mechanism to enhance the accumulation of therapeutic agents within malignant tissue. Upon internalization by macrophages and endothelial cells, MPN could potentially trigger the release of endogenous nitric oxide (NO), a key mediator of vasodilation, in a concentration-, and time-dependent manner. Following MPN administration, raster-scanning optoacoustic mesoscopy (RSOM) revealed vasodilation across multiple tissues, with a strong effect observed in tumors. To assess enhanced tumor accumulation, we injected 70 kDa fluorescent dextran and demonstrated via histology a markedly increased fluorescence signal exclusively in MPN-treated tumors compared to controls 24 h later. In addition, positron emission tomography (PET) imaging of ^89^Zr-labeled Feraheme nanoparticles showed significantly greater tumor accumulation after a 15 min MPN pretreatment. Finally, general serum biochemistry panels and histological analyses of major organs in healthy mice revealed that MPN did not induce observable short-term toxicity under the tested conditions (single or repeated MPN dosing). Overall, this study demonstrates that MPN-induced vasodilation occurring within minutes enhances intratumoral deposition of macromolecules and small nanoparticles. Together with their longer-term effects on phagocytosis inhibition, these findings indicate that MPN can improve therapeutic delivery through complementary, time-dependent mechanisms that increase tumor perfusion and vascular permeability.

## Graphical abstract

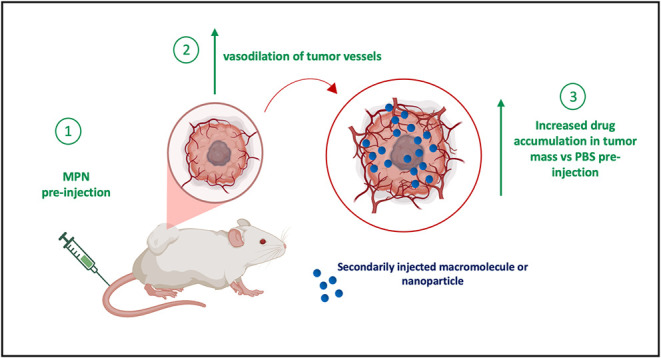

## Introduction

The delivery of macromolecules and nanoparticles to tumors relies on a complex series of processes occurring at both the systemic and tissue levels. At the systemic level, Kupffer cells positioned as sentinels along the vascular lumen of liver sinusoids, splenic macrophages forming a dense filtration network, alveolar macrophages lining the pulmonary microvasculature, and circulating monocytes collectively contribute to the rapid removal of foreign materials, including therapeutic nanoparticles (e.g., nanomedicines).^1–4^ At the tissue level, mass transport from the vascular compartment into the tumor parenchyma is impaired by the dysregulated and abnormal tumor vasculature, which leads to poor blood perfusion and irregular endothelial fenestrations, thereby limiting the intratumoral accumulation of systemically injected therapeutic agents.^5^ Blood flow to tumors is further restricted by the immature vasculature bed,^6^ which is rapidly outgrown by the tumor cells itself. As a result, some vessels collapse under elevated interstitial fluid pressure,^7^ while others remain structurally undeveloped, contributing to increased vascular resistance.^8^ Together, these factors result in reduced and highly heterogeneous perfusion, ultimately limiting the transport of small molecules, macromolecules as well as nanoparticles from the bloodstream into the tumor tissue and their accumulation within the bulk tumor.

In previous work, we demonstrated that systemically administered methyl palmitate nanoparticles (MPN) can transiently and safely suppress the phagocytic activity of macrophages, thereby prolonging the circulation time and enhancing the tumor accumulation of systemically injected nanomedicines.^2^ Different strategies have been recently explored by other groups to improve intratumoral transport of systemically administered therapeutic agents by acting at tumor level. One such approach focuses on remodeling and normalizing the tumor vasculature using antiangiogenic therapies.^9,10^ By partially restoring vascular architecture, these treatments aim to improve tissue perfusion. However, vascular normalization often reduces endothelial fenestration size, which can limit the intratumoral accumulation of most conventional systemically injected nanomedicines.^11^ More recently, immunomodulatory strategies have sought to exploit the tumor immune landscape by activating immunostimulatory cells to combat cancer. These approaches can also indirectly affect the tumor vasculature, through cytokine signaling within the tumor microenvironment.^10^ Although such strategies may enhance vascular function, their effects typically require days to develop, as they depend on extensive cellular and biophysical remodeling of the malignant tissue. More recent efforts have shifted toward strategies that exploit the existing, although abnormal, tumor vasculature to achieve more immediate improvements in blood perfusion, permeability, and subsequent extravasation of therapeutic agents from the vasculature. One such strategy employs focused high-intensity ultrasound in combination with microbubbles to transiently increase vascular permeability, followed by the administration of therapeutic agents.^12^ However, to date, this method has primarily resulted in extravasation and accumulation of nanoparticles *perivascularly,* without a clear increase in tumor perfusion.

Another promising strategy centers on modulating nitric oxide (NO) signaling. NO is a potent mediator of rapid vasodilation^13^ and leveraging this effect could improve the short-term transport of macromolecules and nanoparticles by widening the blood vessels.^13^ Mechanistically, NO-mediated vasodilation of tortuous and dense microvascular networks—comprising arterioles, capillaries, and venules—throughout the tumor mass would be expected to increase tumor perfusion by augmenting blood flow through these aberrant vessels.^14,15^ In addition to increased perfusion, vasodilation may further intensify the already elevated vascular permeability characteristic of tumor vasculature. Tumor vessel hyperpermeability correlates with fainter VE-cadherin expression and increased paracellular space between endothelial cells, compared to normal vasculature.^16^ Collectively, the combination of increased perfusion through widened tortuous microvessels and augmented vascular permeability may facilitate greater extravasation and intratumoral accumulation of therapeutic agents. Seminal work by Maeda and colleagues, pioneers of the enhanced permeability and retention (EPR) effect, has previously demonstrated that NO donors and NO-releasing albumin-based constructs can enhance tumor blood flow and improve drug delivery to tumors.^17,18^ Despite this promise, current NO-based therapies face significant practical challenges. The high chemical reactivity and difficult handling of gaseous NO have led researchers to develop NO donors; however, most existing formulations suffer from limited biocompatibility, suboptimal biodegradation profiles, or unfavorable release kinetics.^19^

To overcome these limitations, we developed an alternative strategy to induce vasodilation possibly by stimulating the release of endogenous NO directly within vascular endothelial cells and macrophages, rather than delivering NO via exogenous agents. This approach is enabled by our methyl palmitate nanoparticles (MPN), which are fabricated from naturally occurring components: albumin, the most abundant serum protein, serves as the structural scaffold; and methyl palmitate, an endogenous lipid, functions as the bioactive payload. In the present study, we investigate an additional and previously unexplored property of MPN: their ability to modulate vascular tone and influence the tumor vascular bed, thereby improving the delivery of systemically injected molecules and nanomedicines. This work is motivated by recent findings identifying methyl palmitate as a potential mediator of vasodilation in retinal tissue,^20^ possibly through NO-dependent mechanisms.^21,22^ Given the absence of prior reports examining vasodilation with nanoparticle-formulated methyl palmitate, we completed initial *in vitro* studies to measure the capacity of MPN to induce NO production in macrophages and endothelial cells. We then assessed the vasodilatory activity of MPN *in vivo* by measuring real-time changes in tumor vessel tone following systemic MPN administration. Finally, we examined whether these vascular effects translated into improved transport and intratumoral deposition of macromolecules and nanoparticles.

## Results

### Physico-Chemical Characterizations of Methyl Palmitate Nanoparticles and Cellular Uptake

Methyl palmitate nanoparticles (MPN) were produced by nanoprecipitation, following a procedure described in our previous publication.^2^ Briefly, an ethanol-based methyl palmitate solution was mixed and sonicated with an albumin solution in PBS. After purification, MPN exhibited a hydrodynamic diameter of ∼200 nm (d = 185 ± 1 nm), a narrow size distribution (PDI = 0.01 ± 0.02), and a negative surface electrostatic potential (ζ = −31.5 ± 0.3), as summarized in Figure 1a. MPN appeared predominantly spheroidal in shape, as documented by the transmission electron microscopy image in Figure 1b, and its inset. Given their morphology and based on observations from previous studies,^23–25^ MPN are expected to be efficiently internalized by resident macrophages. To this end, a fluorescent version of MPN was generated by dispersing lipid-Cy5 molecules with methyl-palmitate in the original ethanol solution. The resulting Cy5-MPN displayed physicochemical features comparable to unlabeled MPN; a detailed physicochemical characterization of Cy5-MPN is available in SI1. To test MPN internalization, bone marrow-derived macrophages (BMDM), RAW264.7 cells, and human umbilical vein endothelial cells (HUVEC) were cultured and incubated with Cy5-MPN for 16 h. Cells were then fixed, stained and observed under a confocal microscope (Figure 1c). Fluorescent MPN were predominantly observed in a perinuclear position across all cell types, suggesting efficient cellular uptake. The intracellular localization of MPN was further confirmed via transmission electron microscopy of BMDM (Figure 1d), following a 4-h incubation with the nanoparticles. Samples were counterstained to visualize both lipid and protein components, which constitute the MPN structure. Discrete, spheroidal electron-dense structures surrounded by a darker boundary were observed exclusively in MPN-treated cells. This darker rim is consistent with an albumin-rich outer layer, as albumin contains sialic acid residues whose carboxyl groups are highlighted by uranyl acetate staining. The preservation of particle shape and their appearance as individual entities, rather than aggregates, suggest that the nanoparticles were internalized shortly before fixation. No such structures were detected in untreated control cells.

**Figure 1:**
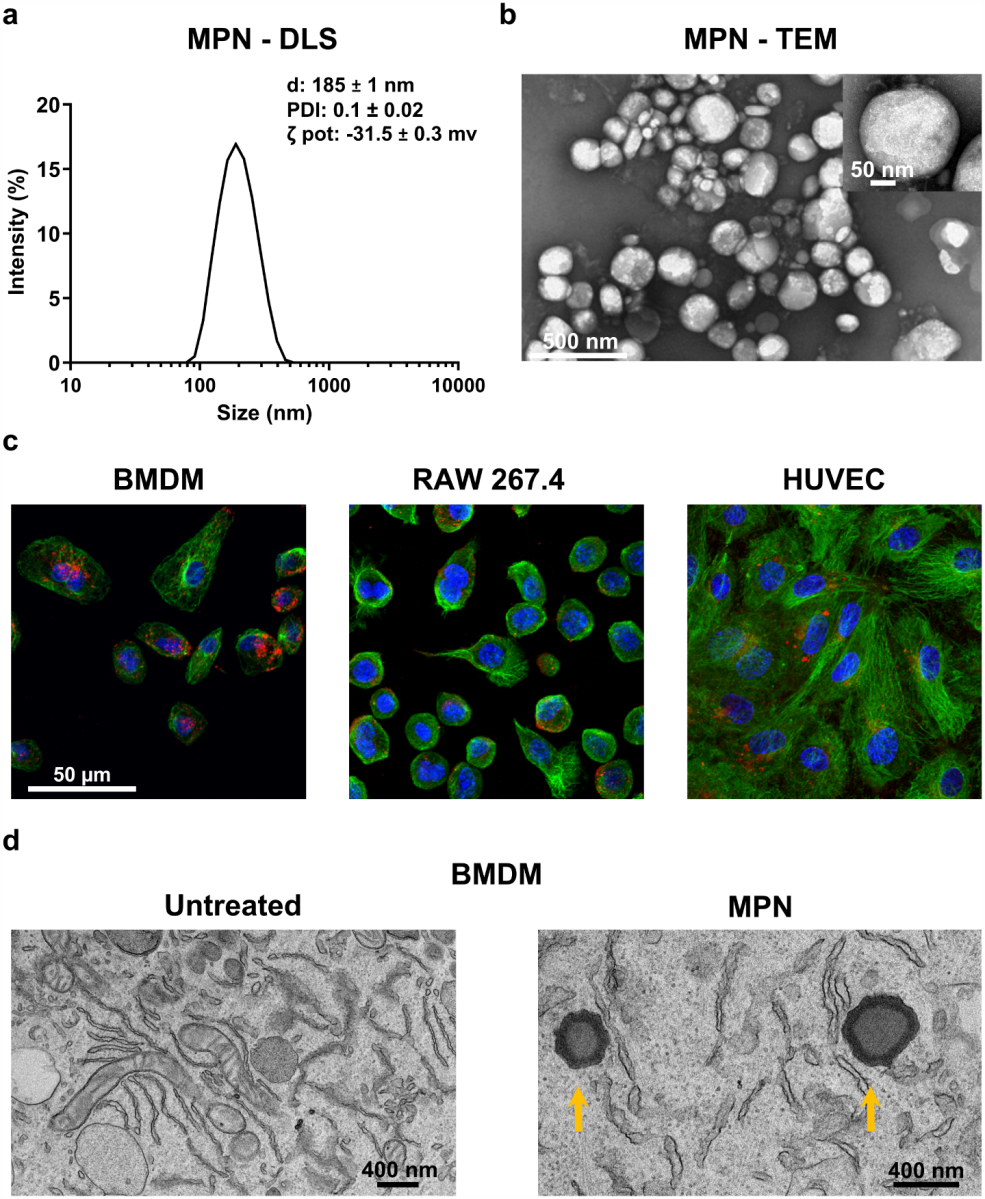
Physicochemical characterization and cellular uptake of MPN. **a.** Dynamic light scattering (DLS) analysis showing the hydrodynamic size distribution of MPN, including diameter (d), polydispersity index (PDI), and surface electrostatic potential (ζ). **b.** Transmission electron microscopy (TEM) images of MPN showing their spheroidal morphology (inset: higher magnification). **c.** Confocal microscopy images demonstrating MPN internalization in BMDM, RAW 264.7 cells, and HUVEC (red: MPN; blue: nuclei; green: tubulin in BMDM and actin in RAW 264.7 cells and HUVEC). **d.** TEM images of BMDM showing internalized MPN (yellow arrows).

### Nitric Oxide Release by Immune and Vascular Cells Exposed to Methyl-Palmitate Nanoparticles

Methyl palmitate was recently identified as a highly potent endogenous vasodilator released by perivascular adipose tissue and retinal tissues, acting on the vessels of the aorta and retina, respectively.^20,21^ The mechanisms underlying its vasoactive function remain debated, but some recent studies related methyl palmitate induced vasodilation to NO release.^26,27^ Given the poor bioavailability of free methyl palmitate and the impracticality of systemic administration of the lipid alone, we investigated whether methyl palmitate nanoparticles (MPN), as carriers of methyl palmitate, could also exert vasodilatory effects. Upon systemic administration, MPN primarily encounter two major cell populations: endothelial cells lining the blood vessel walls and immune cells of the mononuclear phagocyte system (MPS).^28,29^ We hypothesized that exposure to and subsequent internalization of MPN could stimulate NO release in both immune and endothelial cells *in vitro*. To test this hypothesis, Griess assays were performed at predetermined time points on culture media collected from BMDM and HUVEC incubated with MPN. NO release is suspected to occur within minutes from MPN incubation, as shown in Figure 2a–d, which presents the change in nitrite levels over time, for both cell types and varying the MPN concentration from 0 to 0.25 mM equivalent dose of methyl palmitate. Nitrite levels peaked around 10 min and remained relatively stable for up to 1 h. Then, nitrite concentrations gradually declined for the BMDM, while they remained nearly constant for the HUVEC up to 8 h. For both cell types, nitrite production increased in a concentration-dependent manner with increasing MPN dose. Because NO release and synthesis are calcium-dependent processes, we then investigated changes in intracellular calcium concentration in BMDM during the first few minutes after MPN incubation. Results are presented in SI2.

**Figure 2:**
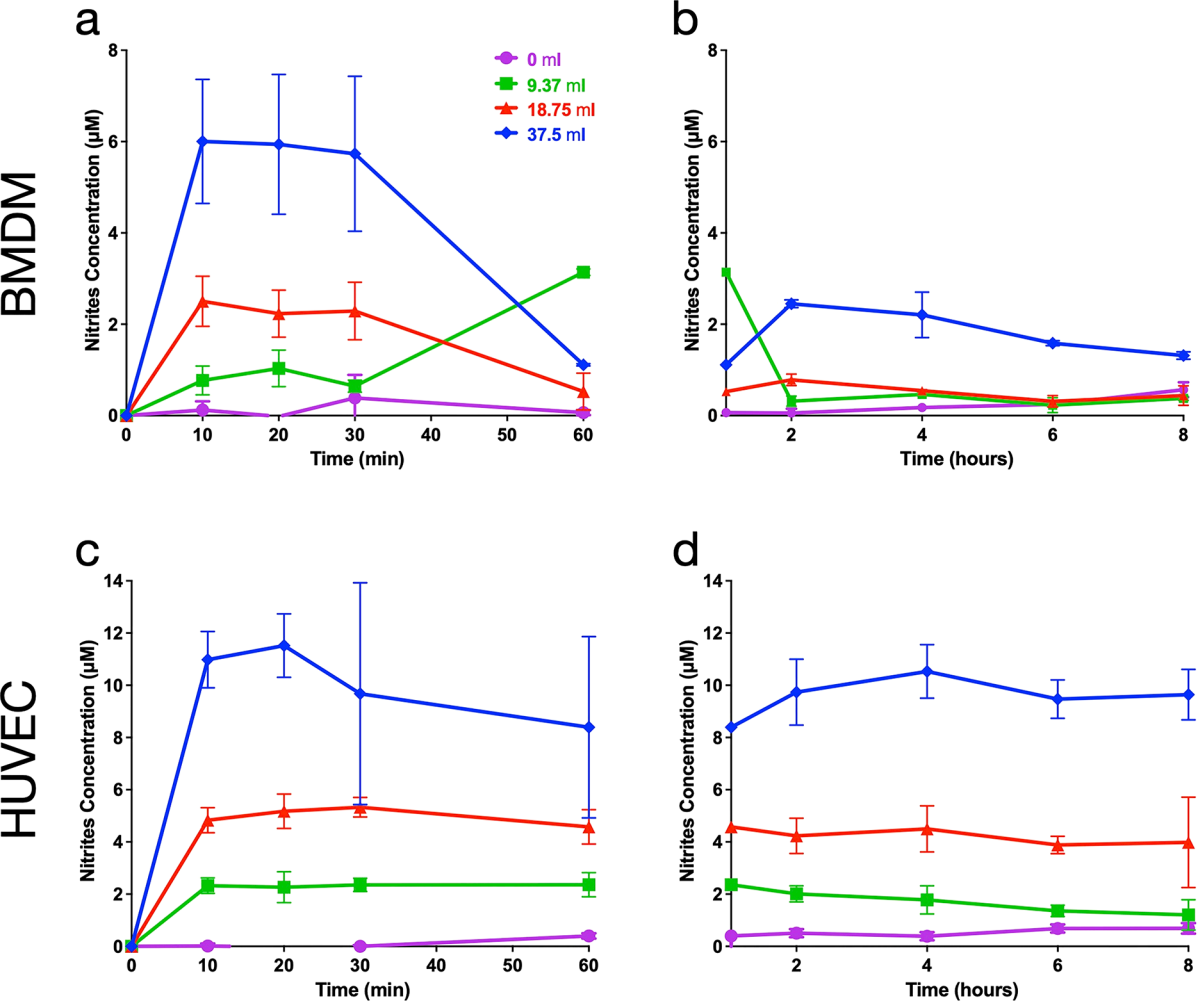
Nitric oxide release and calcium signaling in response to MPN treatment. a–d, Griess assay measuring nitrite accumulation in culture media of BMDM and HUVEC following incubation with increasing doses of MPN at different time points.

### Analysis of Vasodilation in Healthy and Tumor Tissues Induced by Methyl Palmitate Nanoparticles

We next investigated whether methyl palmitate retains its vasoactive function when administered *in vivo* as a nanoformulation. Initial qualitative observations in mice following the systemic administration of MPN revealed a rapid and pronounced vasodilatory response in the ear vasculature of mice, occurring within 5–15 min postinjection (Figure 3a). MPN-injected mice displayed enhanced visibility of blood vessels and fine capillary networks, accompanied by increased ear redness. In contrast, mice injected with an equal volume of the vehicle (PBS), lacked as discernible capillary networks in the ears.

**Figure 3:**
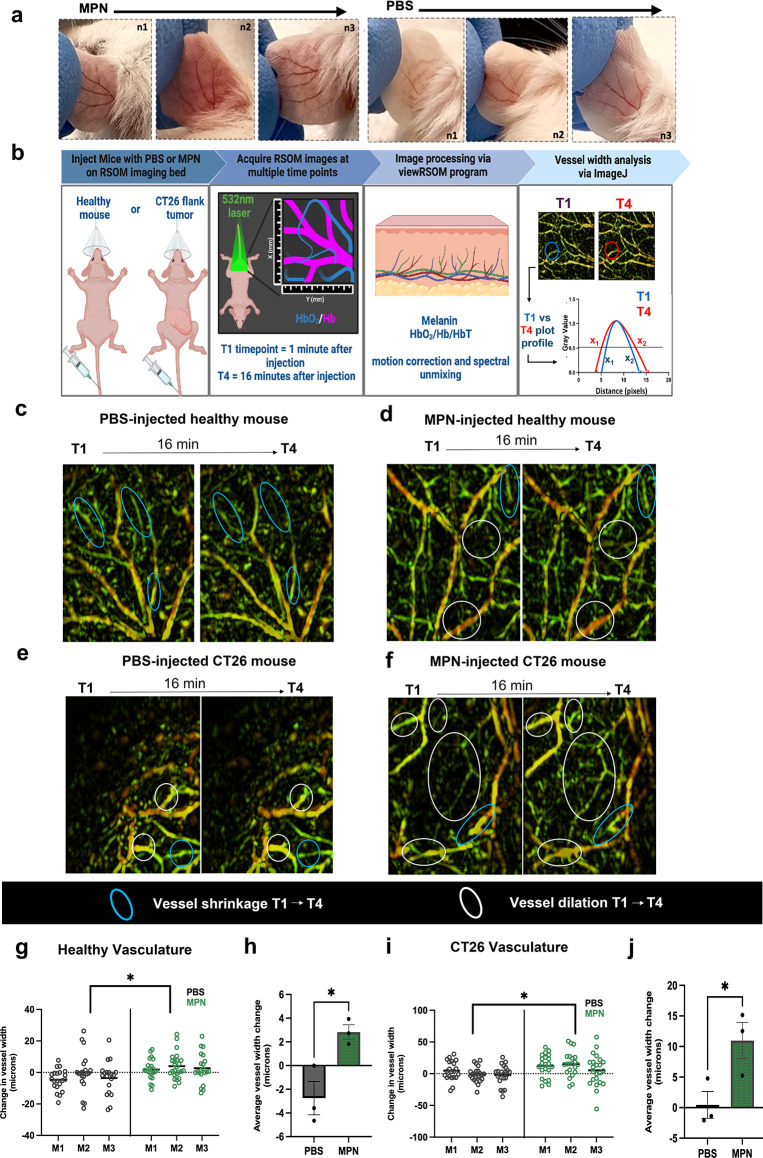
Systemic MPN injection induces varying levels of vasodilation in normal and tumor tissues vasculature. **a,** Pronounced vasodilation and redness is observed in mouse ears within 5–15 min of systemic MPN injection, compared to systemic PBS-injection. **b,** RSOM schematic for vessel width analysis. Nontumor-bearing mice and mice implanted with flank CT26 tumors were anesthetized on the RSOM imaging bed and injected with MPN or PBS. RSOM images of healthy tissue and tumors tissue vasculature were captured 1 min postinjection (T1) up until 16 min postinjection (T4). After motion correction and spectral unmixing of RSOM acquired images, the change in vessel width of approximately 21–25 vessel regions per experimental group (*n* = 3) was analyzed using ImageJ. Representative T1 vs T4 RSOM images of vasculature in **c,** PBS-injected healthy mice **d,** MPN-injected healthy mice **e,** PBS-injected CT26 tumor-bearing mice and **f,** MPN-injected CT26 tumor-bearing mice. (Vessel regions circled in blue represent examples of vessel width reduction that occurred from T1 to T4. Vessel regions circled in white represent examples of vessel width expansion or dilation that occurred from T1 to T4). **g,** Quantification of change in vessel width in approximately 21–25 vessel regions from T1 to T4 in vasculature of healthy mice injected with PBS or MPN (*n* = 3 mice per group, *p* = 0.0249). **h,** Average change in vessel width (μm) of healthy vasculature from T1 to T4 for PBS vs MPN preinjected mice (*p* = 0.022). **i,** Quantification of change in vessel width in approximately 21–25 vessel regions from T1 to T4 in vasculature of CT26 tumor-bearing mice injected with PBS or MPN (*n* = 3 mice per group, *p* = 0.0481). **j.** Average change in vessel width (μm) of CT26 tumor vasculature from T1 to T4 for PBS vs MPN preinjected mice (*n* = 3 mice per group, *p* = 0.0460).

To quantitatively assess vasodilation, we utilized raster scanning optoacoustic mesoscopy (RSOM) to image and analyze the healthy and neoplastic vasculature in response to systemic MPN administration. RSOM is a noninvasive, high-resolution optoacoustic imaging technique that uses laser pulses to excite tissue chromophores, primarily hemoglobin, within blood vessels. The resulting thermoelastic expansion generates ultrasound signals that are detected and reconstructed into detailed vascular images.^30^ For this study, healthy nude mice and nude mice bearing CT26 colon carcinoma flank tumors were intravenously injected with either PBS or MPN, and RSOM images were acquired at short intervals (5 min each) post injection. Four different experimental groups were evaluated: PBS-injected healthy (nontumor-bearing) mice, PBS-injected CT26 tumor-bearing mice, MPN-injected healthy (nontumor-bearing) mice, and MPN-injected CT26 tumor-bearing mice. For all experiments, a dose corresponding to 1.875 mg of methyl palmitate per mouse was administered intravenously. Because MPN are resuspended in PBS, an equivalent volume of PBS was used as a vehicle control injection (negative control). Of note, as previously mentioned, methyl palmitate alone cannot be injected intravenously due to its poor solubility. Nude mice were used for this study to avoid depilation creams in the imaging area, which can cause epidermal irritation, redness, and scarring that interfere with RSOM signal acquisition and vascular visualization.

RSOM images were acquired once before injection, 1 min post injection (T1), and every 5 min up to 20 min postinjection (T2 = 6 min, T3 = 11 min, T4 = 16 min, and T5 = 20 min). Analysis of the postacquisition images revealed that maximal vasodilation occurred approximately 16 min after MPN injection (T4), and this time point was therefore selected for downstream vessel width analysis. Figure 3b summarizes the steps of the RSOM experiment for quantifying vessel width changes in tumor and healthy tissues after mice were injected with PBS or MPN. Vessels were color-coded according to the frequency of the detected ultrasound signal, with larger and deeper vessels (33 – 99 MHz) appearing red, and smaller, more superficial vessels (11–33 MHz) appearing green.^31^ Final images were presented as maximum intensity projections (MIP) composed of merged red and green frequency sub-bands, resulting in composite green, yellow, and orange vascular structures that represent total hemoglobin content in the imaged region.

For vessel width quantification, 21–25 individual vessel regions were randomly selected from blinded images for each experimental group (n = 3 mice per group). The vessel widths at 16 min post injection (T4) were then directly compared to those of the same vessels at 1 min post injection (T1). ImageJ was used to generate T1 and T4 image stacks and perform vessel width measurements, from which the change in vessel width between T1 and T4 was calculated for each vessel as described in the Materials and Methods section. In healthy mice, the vessel width increased to a greater extent between T1 and T4 in mice injected with MPN compared to those receiving PBS injection (Figure 3c, d, and g). Although ∼21–25 vessel regions were selected over the entire scan volume for each mouse in each of the four treatment conditions for quantitative analysis, Figure 3c–f displays RSOM images from one representative mouse per group with 3–5 vessels highlighted with blue or white circles, signifying vessel shrinkage or dilation, respectively. SI3 and SI4 contain T1 and T4 RSOM images acquired from n = 3 mice for each experimental group. In healthy mice that received the MPN injection, a 2.83 μm increase averaged across the three mice, occurred between T1 and T4 (Figure 3h). In contrast, in mice injected with PBS, the vessel analysis indicated a 2.75 μm *reduction* in width, occurring between T1 and T4 (Figure 3h). One explanation for this observation is that in PBS injected mice, an initial volumetric expansion in vessel width 1 min postinjection (T1) occurs, which dissipates at 16 min (T4) post injection, resulting in the calculation of a negative vessel width change (reduction) between T4 and T1. It is also possible that reactive vasoconstriction could have occurred at the later time point in response to mechanical stretch from the injected volume of 300 μL, as might be expected with healthy, structurally normal vasculature. In MPN-injected healthy mice, there may also have been an initial volumetric expansion post injection at the T1 time point, but the sustained vasodilatory effect of MPN at T4 likely counteracts this reactive vasoconstriction, resulting in a net positive change in vessel width, albeit with slightly varied responses among different vessel regions due to heterogeneous biological responses of the vasculature to MPN.

A similar but more pronounced effect was observed in CT26 tumor-bearing mice. Tumor vessels in MPN-injected animals displayed greater vasodilation than those in PBS-injected controls (Figure 3e, f, and i). The average vessel width change between T1 and T4 was +10.98 μm on average in n = 3 MPN-injected mice, while PBS-injected mice (n = 3) displayed a +0.44 μm average increase (Figure 3j). Interestingly, MPN injection in both the healthy and tumor vasculature resulted in the appearance or improved detectability of vessels at time point T4, that were too difficult to capture via RSOM imaging at time point T1. Some striking examples are identified with white circles in Figures 3d and 3f. These vessels could not be quantified with the method used for T4 vs T1 vessel width analysis, as detectable pixels are needed at each time point to calculate a width change. This observation is therefore qualitative and preliminary in nature and warrants further investigation with the development of analytical methods that more holistically quantitate vascular changes at each time point. Notably, MPN-mediated vasodilation appeared more pronounced in tumor vasculature than in healthy skin vasculature in this model, with an approximately greater than 3-fold increase in mean vessel width observed in tumor tissue compared to nontumor tissue (10.97 μm versus 2.83 μm, respectively). While this observation is based on a single tumor model (CT26) and normal tissue comparison (healthy murine skin), it raises the possibility that aberrant vascular structures in tumors respond differently to MPN-mediated vasodilation than structurally normal vasculature. Future studies incorporating multiple tumor models and additional normal tissue comparisons will be necessary to determine the consistency and generalizability of this observation.

### MPN-Induced Vasodilation Enhances the Intratumoral Deposition of Macromolecules

After demonstrating that MPN induce tumor vessel dilation, we next investigated whether this vascular priming effect could lead to a measurable increase in the intratumoral accumulation of macromolecules when administered systemically after MPN injection. To this end, CT26 tumor-bearing mice were first injected intravenously with MPN and, 15–20 min later, retro-orbitally injected with 70 kDa fluorescent dextran, as outlined in Figure 4a. Twenty-4 h after injection, mice were sacrificed, tumors were explanted, and tissue sections were prepared for fluorescence analysis. Fluorescence microscopy imaging was used to compare dextran accumulation (green signal) within tumors from mice which were pretreated (primed) with PBS or MPN. Quantitative analysis was performed by measuring the total tumor slice area and the fraction of that area positive for dextran fluorescence. In Figure 4b, two CT26 tumor sections showing a clear difference were reported, one for the PBS injection group and one for the MPN pretreated group. To provide a wider view on the plotted data, 8 more randomly picked additional slices were reported in SI5. For each section, the full area used for quantification is shown on the left, and the dextran-positive area is shown on the right. By comparing the positive areas of the slices of the two groups (PBS and MPN), a greater extent of fluorescent dextran accumulation is clearly observed in tumors from mice pretreated with MPN. Images were false-colored to further highlight the difference in dextran fluorescence intensity and topographic distribution within the CT26 tumors (Figure 4c). To compare dextran accumulation between PBS and MPN priming conditions, the dextran-positive area fraction of each slice was quantified. A statistically significant difference was observed between the two groups, with slices from MPN pretreated mice showing approximately 1.5-fold larger dextran-positive areas. With the aim of furtherly study dextran distribution inside tumors, radial profiles were analyzed on 8 different slices, revealing an overall superior fluorescence intensity in MPN pretreated mice Figure 4e. Moreover, to have information on signal topography, dextran fluorescence intensity was measured in 5 concentric areas of the slices (center; intermediate 1; intermediate 2; intermediate 3; periphery). Significant differences in fluorescence intensity measures (PBS vs MPN) were found in the first 3 areas, in favor of MPN treated mice (P = 0.038; P= 0.024; P= 0.035); results are reported in SI6 and the ratio of the intensities found in the central areas and the peripheral ones were reported in Figure 4f. These analyses revealed that in MPN injected mice, 70 kDa dextran was more present in the central areas of the tumor. In contrast, in the PBS pretreated mice, a slightly superior accumulation (although not significantly different from MPN treated mice) was found at the periphery. These results indicate that short-term MPN priming is effective in enhancing macromolecule accumulation within solid tumors. Taken together, the data presented in Figures 3 and 4 indicate that the transient and reversible vasodilation induced by MPN promotes enhanced intratumoral accumulation and retention of macromolecules (such as 70 kDa dextran) for at least 24 h postinjection.

**Figure 4:**
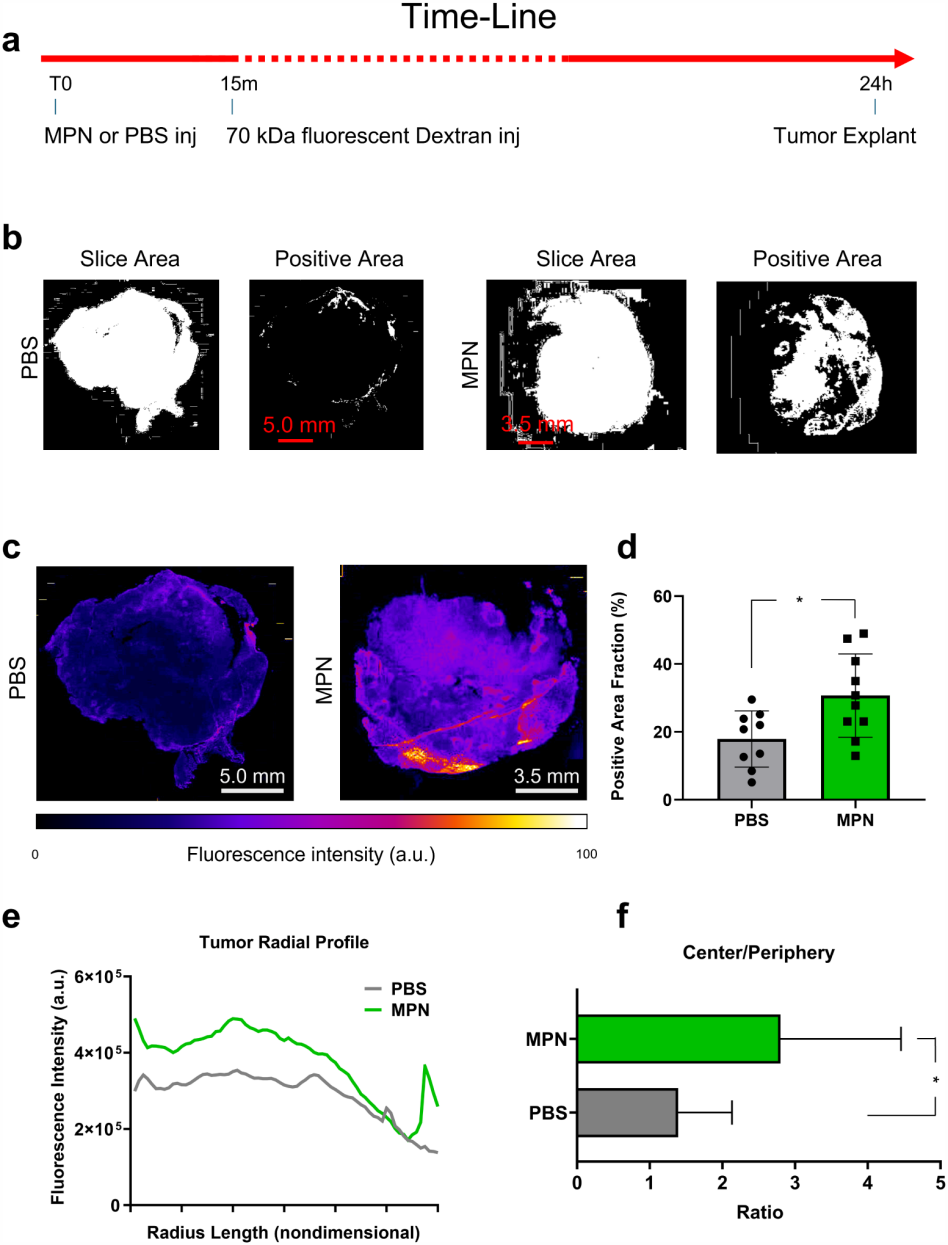
Quantification of intratumoral dextran accumulation. **a,** Timeline of treatments and procedures in CT26 tumor-bearing mice. **b,** Representative tumor slice area (left) and dextran-positive area (right) in PBS-injected and MPN-pretreated mice. **c,** False-colored tumor images showing spatial distribution and intensity of dextran fluorescence in PBS-DEX and MPN-DEX mice. **d,** Quantification of dextran-positive area fraction in tumor slices from PBS-DEX and MPN-DEX groups (*p* = 0.018). e, Tumor radial profile (average *n* = 8). **d,** Center/Periphery ratio (*n* = 8, *p* = 0,039).

### MPN-Induced Vasodilation Enhances the Intratumoral Deposition of Nanoparticles

We next tested whether MPN vascular priming could also improve the accumulation of the FDA-approved iron oxide nanodrug Feraheme (FH). FH is a 750 kDa ultrasmall superparamagnetic iron oxide nanoparticle, with a colloidal diameter ranging from 17 to 31 nm and is used clinically for the treatment of iron-deficiency anemia. More recently, FH has attracted interest as an anticancer agent due to its ability to generate reactive oxygen species (ROS) and induce oxidative stress-mediated cancer cell death.^32^ In addition, FH has been reported to induce a pro-inflammatory phenotype in macrophages, supporting its potential use as an immune-stimulating antitumor adjuvant.^33,34^ FH can also be noncovalently loaded with various therapeutic small molecules, such as doxorubicin, enabling effective nanoparticle-based delivery of small molecules to tumors.^35^ Given its broad range of applications in cancer therapy, strategies that enhance FH accumulation within tumor masses could further improve its therapeutic potential as an anticancer agent.

For this study, chelator-free radiolabeling of FH with the positron-emitting radionuclide ^89^Zirconium (^89^Zr-FH) was completed. Chelator-free radiolabeling of FH has previously been used to enable PET/CT-based *in vivo* visualization, tracking, and quantification of FH nanoparticle biodistribution in tumor and nontumor tissues, without altering the physiochemical properties of the nanoparticles.^36,37^ Following existing protocols,^36^ pH neutralized ^89^Zr was incubated with FH nanoparticles at 95 °C for 2 h, after which deferoxamine (DFO) was used to chelate unbound radionuclide, followed by Amicon filtration of ^89^Zr-FH. This process yielded a radiolabeling efficiency exceeding 90%, with instant thin-layer chromatography indicating that 91.5% ± 9% (n = 3) of the radioactivity was bound to the nanoparticle (Figure 5a).

**Figure 5:**
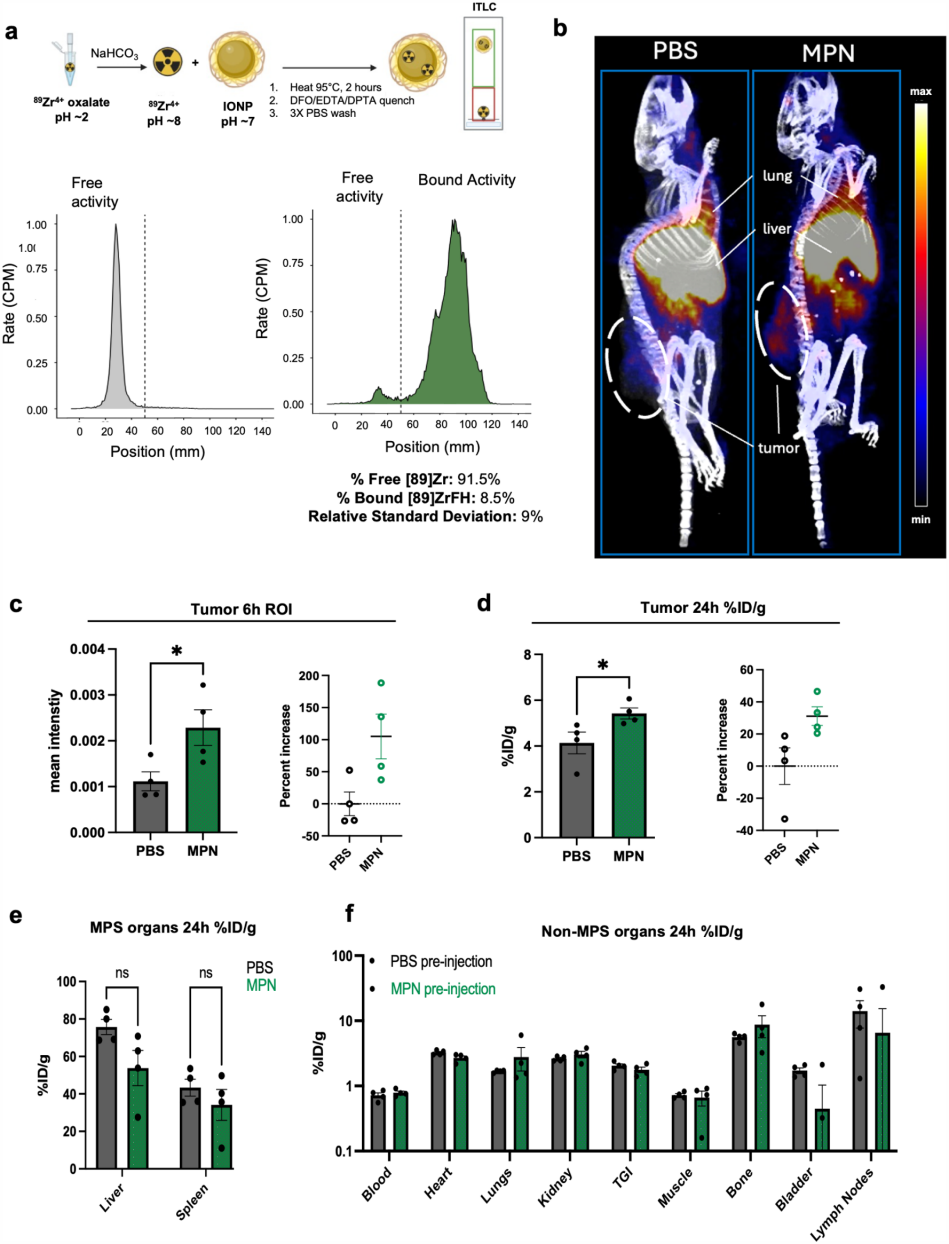
Quantification of intratumoral Feraheme (FH) accumulation. **a,** schematic of chelator free radiolabeling. FH is added to ^89^Zr-oxalate after neutralization with sodium bicarbonate to a pH of ∼7–8. The solution is then heated to 95 °C for 2 h, after which unbound ^89^Zr is chelated with deferoxamine (DFO), followed by PBS washes in a 30 kDa Amicon filtration unit. To check radiolabeling efficiency, radiolabeled nanoparticle is run on an ITLC strip where free activity stays at the origin (corresponding to a large peak before the 50 mm position on the ITLC strip) and radioactivity bound to the FH nanoparticle travels up the strip with the particle (corresponding to a large peak above 50 mm and a small free activity peak below the 50 mm position on the ITLC strip). **b,** PET/CT image displaying ^89^Zr-FH biodistribution 6 h (h) after injection. The tumor mass is circled with a dashed line and tissues with substantial nanoparticle uptake are labeled. **c,** 6 h quantification of ^89^Zr-FH in tumor tissue (region of interest ROI) displayed as mean intensity (*p* = 0.0376) and percent increase compared to PBS-preinjected mice. **d,** 24 h analysis of % injected dose of ^89^Zr-FH per gram of tissue (%ID/g) in tumors (*p* = 0.0286). Percent increase compared to PBS-preinjected mice also displayed. **e,** Quantification of ^89^Zr-FH in macrophage-high organs of the mononuclear phagocyte system (MPS), displayed as %ID/g, at 24 h post injection. **f,** Quantification of ^89^Zr-FH displayed as %ID/g, at 24 h post ^89^Zr-FH injection in tissues with lower macrophage content compared to the liver and spleen. TGI= total gastrointestinal tract.

*In vivo* biodistribution studies were then completed to determine if MPN preinjection (*vascular priming*) could improve the accumulation of ^89^Zr-FH in CT26 tumor masses, compared to PBS (control) preinjection. Mice were injected with 100 μL of ^89^Zr-FH, 15 to 20 min post MPN priming or PBS injection. PET/CT imaging was performed 6 h postinjection using a Siemens Inveon scanner to evaluate whole-body biodistribution. Images were reconstructed as maximum intensity projections (MIP) using Inveon Research Workplace software. As expected, ^89^Zr-FH exhibited typical nanoparticle biodistribution, with prominent signal in the lungs and liver (Figure 5b). PET/CT images of all animals (n = 4 mice per PBS or MPN preinjection group) in this study are in SI7. Quantitative region-of-interest analysis of PET images revealed an approximately 2-fold (∼100%) increase in tumor-associated ^89^Zr-FH signal in MPN-pretreated mice compared with PBS-pretreated controls at 6 h postinjection (n = 4 mice per group; Figure 5c).

Mice were subsequently sacrificed 24 h after ^89^Zr-FH injection, and organs were harvested for gamma counting to determine percent injected dose per gram of tissue (%ID/g). Tumors from MPN-pretreated mice exhibited a sustained ∼30% increase in ^89^Zr-FH accumulation compared with PBS controls at 24 h (Figure 5d), indicating prolonged retention following enhanced early deposition.

Accumulation of ^89^Zr-FH in the liver and spleen was also evaluated, as these organs are part of the MPS and contain high levels of tissue resident macrophages. MPN preinjection modestly reduced liver accumulation (not statistically significant) while increasing tumor accumulation of the administered agent, although the remaining redistributed signal may reside in unmeasured compartments (Figure 5c-e). A slight trend toward reduced splenic FH accumulation was also observed following MPN pretreatment, though this too did not reach statistical significance (Figure 5e). These results are consistent with the relatively minor vasodilatory effects of MPN in healthy tissues and with the short (15 min) pretreatment interval used in this study, which is insufficient to induce macrophage phagocytic suppression in the liver (previously observed at ∼4 h).^2^

Similarly, no significant differences in ^89^Zr-FH accumulation were detected in organs with lower macrophage density (Figure 5f). These findings align with RSOM analyses demonstrating that MPN elicits a differential vasodilatory response in CT26 tumor vasculature compared to healthy tissue vasculature. This vascular response in CT26 tumors likely reduces interstitial fluid pressure and enhances tumor perfusion through increased blood flow within vasodilated tortuous vessels. Additionally, vascular permeability likely increases, through widening of paracellular spaces between endothelial cells, collectively facilitating increased nanodrug extravasation and accumulation within the malignant tissue.

Collectively, these results highlight the importance of pretreatment timing in exploiting the distinct biological effects of MPN. While longer pretreatment durations (∼4 h) was previously reported to suppress macrophage uptake at the systemic level and favor delivery of larger nanoparticles (≥200 nm),^2^ the shorter 15 min pretreatment used here preferentially enhances tumor perfusion, improving the accumulation of smaller nanoparticles (e.g., the 17–30 nm FH nanoparticles) and macromolecules (e.g., 70 kDa dextran).

### Toxicity Tests on Healthy Methyl Palmitate Nanoparticles Treated Mice

To comprehensively evaluate the tolerability of MPN, we performed a series of toxicity assessments in healthy mice. First, we analyzed serological blood parameters in C57BL/6 mice that received a single high dose of MPN corresponding to 3.75 mg of methyl palmitate per 20 g body weight, administered via tail-vein injection. Blood samples were collected 24 h after treatment. All measured serum chemistry parameters are summarized in Table 1 and graphically represented in SI8. No statistically significant alterations were detected in any parameter except for uric acid, which was significantly reduced in MPN-treated mice compared with controls (Table 1). This reduction is consistent with increased nitric oxide (NO) production, as uric acid is a known endogenous scavenger of NO by acting as a compensative mechanism aimed to reduce oxidative stress; unlike elevated uric acid levels, this decrease is not considered indicative of toxicity.^38–41^ Hematological parameters measured under the same conditions likewise showed no significant differences between control and MPN pretreated mice (Table 2 and SI9).

**Table 1:** Serological Blood-Panel Parameters of Mice which Underwent One Single Injection of a High Dose of MPN Equivalent to 3.75 Mg

		CTRL	MP
Albumin	g/dl	2.90 ± 0.14	2.94 ± 0.18
Creatinine	mg/dl	0.28 ± 0.02	0.26 ± 0.03
Amylase	U/L	1069.60 ± 31.15	1096.80 ± 261.07
Aspartate aminotransferase	U/L	122.60 ± 32.93	120.80 ± 45.74
Alanine Aminotransferase	U/L	40.40 ± 9.34	47.20 ± 27.50
Alkaline Phosphatase	U/L	140.80 ± 12.56	139.80 ± 22.08
Glucose Hexokinase	mg/dl	262.00 ± 22.11	257.40 ± 16.77
Urea	mg/dl	64.60 ± 6.35	61.00 ± 10.86
**Uric Acid**	**mg/dl**	**1.44 ± 0.48**	**0.62 ± 0.31**
Triglycerides	mg/dl	105.40 ± 15.44	101.80 ± 36.21
Cholesterol	mg/dl	62.80 ± 5.26	63.60 ± 3.41
Lactate Dehydrogenase	U/L	514.40 ± 133.51	756.00 ± 255.58
Creatin Kinase	U/L	1355.20 ± 926.63	1277.40 ± 698.94
Iron	μg/dl	132.80 ± 17.22	115.20 ± 26.01
Serum Cholinesterase	U/L	3412.00 ± 210.81	3395.20 ± 390.55
Calcium	mg/dl	8.66 ± 0.56	8.34 ± 0.48
Phosphate	mg/dL	6.61 ± 0.39	6.68 ± 0.61
LDL Cholesterol	mg/dL	16.20 ± 2.68	14.40 ± 1.95

**Table 2:** Hematologic Panel Parameters of Mice which Underwent One Single Injection of a High Dose of MPN Equivalent to 3.75 mg

		CTRL	MP
RBC	M/μL	10.03 ± 0.36	9.54 ± 0.46
HGB	g/dL	14.70 ± 0.55	13.98 ± 0.57
HCT (%)	%	48.44 ± 2.17	45.58 ± 2.36
MCV (fL)	fL	48.28 ± 0.43	47.80 ± 0.23
MCH (pg)	pg	14.66 ± 0.05	14.68 ± 0.15
MCHC (g/dL)	g/dL	30.34 ± 0.27	30.66 ± 0.40
RDW-SD (fL)	fL	27.34 ± 1.00	26.16 ± 0.32
RET (K/μL)	K/μL	422.70 ± 28.22	421.76 ± 40.63
RET (%)	%	4.22 ± 0.26	4.41 ± 0.23
PLT (K/μL)	K/μL	924.00 ± 229.21	1002.80 ± 134.16
WBC (K/μL)	K/μL	8.89 ± 1.82	10.30 ± 1.58
NEUT (K/μL)	K/μL	1.30 ± 0.65	0.98 ± 0.18
LYMPH (K/μL)	K/μL	7.56 ± 1.04	8.96 ± 1.42
MONO (K/μL)	K/μL	0.14 ± 0.06	0.20 ± 0.06
EO (K/μL)	K/μL	0.15 ± 0.04	0.16 ± 0.02
BASO (K/μL)	K/μL	0.01 ± 0.01	0.00 ± 0.01
NEUT (%)	%	13.30 ± 4.67	9.50 ± 1.13
LYMPH (%)	%	86.06 ± 7.14	86.94 ± 1.68
MONO (%)	%	1.56 ± 0,43	2.00 ± 0.55
EO (%)	%	1.68 ± 0.42	1.52 ± 0.11
BASO (%)	%	0.06 ± 0.05	0.04 ± 0.05
RET-He (pg)	pg	18.48 ± 0.16	18.24 ± 0.18

To assess tolerability under repeated dosing, a separate cohort of mice received four injections of MPN administered twice per week over a 2-week period at a lower dose equivalent to 0.94 mg of methyl palmitate per 20 g body weight. Blood samples were collected 24 h after the final injection, and serum chemistry results are listed in Table 3 and SI10. Overall, no major abnormalities were observed. A mild increase in transaminases and serum cholinesterase was detected in MPN-treated mice; however, these changes did not reach statistical significance. A small but statistically significant increase in lactate dehydrogenase (LDH) was observed, although this elevation is not typically associated with clinical toxicity. All other parameters remained within physiological reference ranges. In contrast to the acute high-dose condition, uric acid levels in the repeated low-dose cohort showed a slight, nonsignificant increase, suggesting that higher MPN doses may be required to induce sufficient NO production to measurably reduce uric acid at the analyzed time point. Consistent with these findings, hematological analysis revealed no significant differences between MPN pretreated and control mice under repeated dosing conditions (Table 4 and SI11).

**Table 3:** Serological Blood-Panel Parameters of Mice which Underwent 2 Injections per Week with a Lower Dose, Equivalent to 0.94 mg of Methyl Palmitate per Mouse (20 g)

		CTRL	MP
Albumin	g/dl	1.30 ± 0.20	1,60 ± 0,10
Creatinine	mg/dl	0.32 ± 0.06	0,35 ± 0,03
Amylase	U/L	857.33 ± 86.70	1082,00 ± 40,26
Aspartate aminotransferase	U/L	61.33 ± 5.03	152,33 ± 92,72
Alanine Aminotransferase	U/L	18.33 ± 2.08	55.33 ± 100.20
Alkaline Phosphatase	U/L	53.67 ± 12.50	97.00 ± 23.52
Glucose Hexokinase	mg/dl	200.00 ± 22.91	252.00 ± 27.06
Urea	mg/dl	62.00 ± 6.00	59.33 ± 7.51
Uric Acid	mg/dl	0.43 ± 0.06	0.63 ± 0.15
Triglycerides	mg/dl	155.00 ± 22.07	149,67 ± 50,54
Cholesterol	mg/dl	47.67 ± 1.15	71,33 ± 3,21
Lactate Dehydrogenase	U/L	329.67 ± 71.45	520.67 ± 74.10
Creatin Kinase	U/L	580.33 ± 125.24	690.67 ± 506.32
Iron	μg/dl	102.33 ± 9.07	142.33 ± 15.18
Serum Cholinesterase	U/L	2897.33 ± 299.54	3874.00 ± 640.11
Calcium	mg/dl	6.70 ± 0.10	6.03 ± 0.64
Phosphate	mg/dL	6.19 ± 0.56	4.74 ± 0.58
LDL Cholesterol	mg/dL	11.00 ± 1.00	12.33 ± 3.51

**Table 4:** Hematologic Panel Parameters of Mice which Underwent 2 Injections per Week with a Lower Dose, Equivalent to 0.94 mg of Methyl Palmitate per Mouse (20 g)

		CTRL	MP
RBC	M/μL	8.73 ± 0.31	9.54 ± 0.46
HGB	g/dL	12.60 ± 0.44	13.98 ± 0.57
HCT (%)	%	44.53 ± 2.46	45.58 ± 2.36
MCV (fL)	fL	51.00 ± 2.07	47.80 ± 0.23
MCH (pg)	pg	14.43 ± 0.15	14.68 ± 0.15
MCHC (g/dL)	g/dL	28.33 ± 0.81	30.66 ± 0.40
RDW-SD (fL)	fL	31.87 ± 1.59	26.16 ± 0.32
RET (K/μL)	K/μL	315.13 ± 49.03	421.76 ± 40.63
RET (%)	%	3.62 ± 0.65	4.41 ± 0.23
PLT (K/μL)	K/μL	966.67 ± 182.66	1002.80 ± 134.16
WBC (K/μL)	K/μL	7.15 ± 1.00	10.30 ± 1.58
NEUT (K/μL)	K/μL	0.84 ± 0.16	0.98 ± 0.18
LYMPH (K/μL)	K/μL	6.04 ± 1.03	8.96 ± 1.42
MONO (K/μL)	K/μL	0.17 ± 0.04	0.20 ± 0.06
EO (K/μL)	K/μL	0.10 ± 0.01	0.16 ± 0.02
BASO (K/μL)	K/μL	0.00 ± 0.00	0.00 ± 0.01
NEUT (%)	%	11.90 ± 2.60	9.50 ± 1.13
LYMPH (%)	%	84.20 ± 3.32	86.94 ± 1.68
MONO (%)	%	2.40 ± 0.95	5.17 ± 3.63
EO (%)	%	1.50 ± 0.20	2.03 ± 0.75
BASO (%)	%	0.00 ± 0.00	0.03 ± 0.06
RET-He (pg)	pg	18.30 ± 0.36	18.47 ± 0.31

To further evaluate potential tissue-level toxicity, additional mice were treated under the same dosing regimens used for serum and hematological analyses. Twenty-4 h after a single high-dose MPN injection, or 24 h after the final injection in the repeated low-dose cohort, mice were sacrificed and major organs were harvested for histological examination. Hematoxylin and eosin (H&E) staining of liver, spleen, lung, kidney, and heart sections revealed no observable pathological abnormalities in mice treated with either a single high dose (3.75 mg methyl palmitate per 20 g body weight) or repeated low-dose injections (0.94 mg per 20 g body weight), compared with untreated controls (Figure 6a,b). Collectively, these results indicate that MPN did not induce observable short-term toxicity under the tested conditions (following both acute and short-term repeated administration).

**Figure 6:**
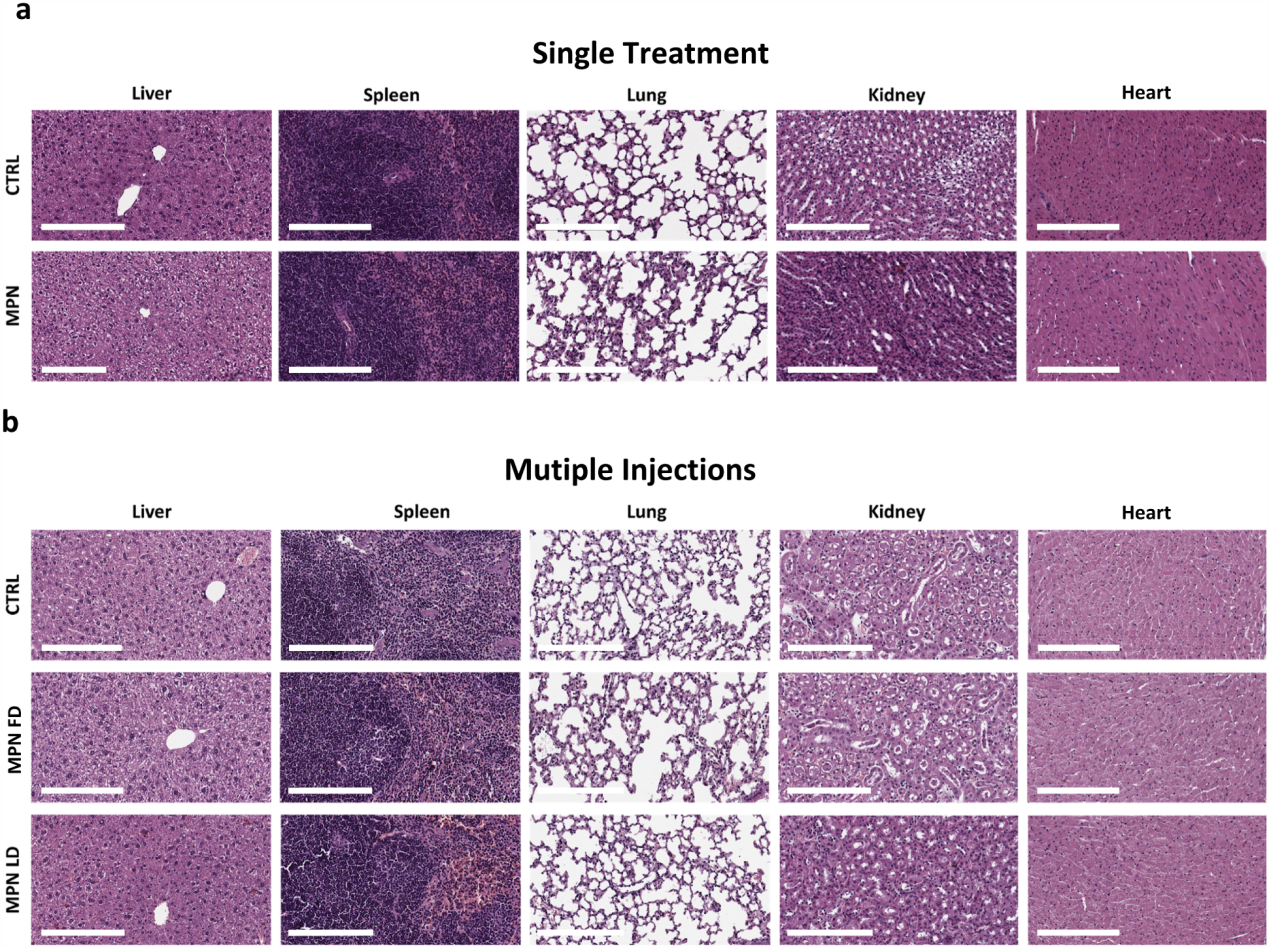
Main organs histology in MPN treated healthy mice. **a,** Representative H&E-stained sections of major organs (liver, spleen, lung, kidney, and heart) from control mice and mice treated with a single high dose of MPN (3.75 mg methyl palmitate per 20 g body weight); scale bars = 200 μm. **b,** Representative H&E-stained sections of major organs from mice treated with repeated MPN injections (twice per week for 2 weeks) at either full dose (FD= 3.75 mg per 20 g) or low dose (LD= 0.94 mg per 20 g); scale bars = 200 μm. No overt histopathological abnormalities were observed.

## Discussion

In the present paper a novel strategy to improve macromolecules and nanoparticles delivery at tumor tissue by acting on the vascular tone is presented. The strategy relies on the quick vasodilation observed *in vivo,* minutes after MPN systemic injection. It is the authors’ opinion that the mechanism behind the vasoactive function of MPN might be mediated by NO release directly from endothelial cells and macrophages upon MPN internalization. The involvement of NO is proposed as a plausible mechanism based on: (i) the existing literature showing that methyl palmitate can stimulate NO production;^26,27^ *in vitro* data demonstrating increased nitrite levels in macrophages and endothelial cells culturing media starting minutes after MPN treatment (Figure 2a–d); (iii) *in vivo* observations such as the reduction in plasma uric acid (Table 1), a compensative mechanism aimed to reduce oxidative stress, that recent studies correlate to NO overproduction.^41^ While the relative contribution of different cell populations to NO production cannot be established *in vivo* based on the current data, considering the physicochemical properties and known biodistribution of MPN, we hypothesize that macrophages, particularly Kupffer cells in the liver, represent a major contributor to NO production.^2^ This hypothesis is supported by two of the following points: (i) nanoparticle–cell interactions are driven by physical contact, and particles of ∼200 nm diameter are generally characterized by limited margination toward the endothelial wall, resulting in reduced interaction with endothelial cells compared to circulating phagocytic cells;^42,43^ (ii) nanoparticles of this size, including MPN, are known to accumulate predominantly in the liver, where Kupffer cells serve as the primary clearance system and efficiently internalize most circulating nanomaterials.^2^ It is established that endothelial cells, and other components of the mononuclear phagocyte system, including splenic macrophages and circulating monocytes, may also contribute to NO production. For human umbilical vein endothelial cells this is also supported by our in vitro data of Figure 2c–d. Therefore, authors cannot exclude a combined contribution from multiple cell types. Despite that the cellular source of NO *in vivo* remains to be fully elucidated, macrophages could represent the major, but not the exclusive contributor.

MPN vasodilation effect was found to be more prominent in CT26 tumor tissue rather than in healthy tissue and this finding correlates with the superior 24 h accumulation of both macromolecules and FH. While for macromolecules, this response might be depending by the sole vasodilation, authors cannot exclude FH accumulation at the tumor site is likely not exclusively attributable to vasodilation, and that additional factors, such as altered systemic distribution, circulation time, and modulation of MPS uptake, may also contribute. In particular, in our previous work,^2^ the inhibition of phagocytosis by MPN was found to be able to influence the biodistribution of secondary nanoparticles by prolonging their circulation time and altering organ uptake. In our previous work, with 200 nm spherical polymeric nanoparticles (and other particles), this inhibition was shown to peak approximately 4 h after MPN administration, whereas in the present study the 30 nm FH were injected 15–20 min after MPN pretreatment. Given that FH has a relatively long circulation half-life (∼15 h),^44^ phagocytosis inhibition could potentially contribute to nanoparticle accumulation at later time points. To better understand the relative contributions of these mechanisms, authors compared FH tumor accumulation at 6 and 24 h postinjection. The observation of a higher relative increase at the earlier time points suggests that short-term processes, such as MPN-induced vasodilation, which occurs within minutes after administration, may play a dominant role in the initial enhancement of tumor uptake. At later time points, FH appears to be partially retained rather than progressively accumulating, which would be expected if prolonged circulation due to phagocytosis inhibition were the primary driver. That said, authors cannot exclude a combined contribution of multiple mechanisms.

Authors would like to highlight the fact that MPN were then found to be capable to induce two distinct biological effects (i.e.; vasodilation and inhibition of phagocytosis) that occur on different time scales and may be selectively leveraged by adjusting the timing of the pretreatment (approximately 15 min and ∼4 h, respectively), depending on the physicochemical properties of the secondary nanoparticles. In particular, nanoparticles with smaller hydrodynamic diameters (such as FH) are more likely to benefit from the early vasodilation effect, whereas larger nanoparticles (∼200 nm) may benefit more from reduced phagocytic clearance of MPS at later time points. That said, these mechanisms are surely not mutually exclusive, and authors cannot avoid considering that both processes may contribute, to some extent, to the observed increase in tumor accumulation under short pretreatment conditions. However, as mentioned above, the contribution of phagocytosis inhibition at early time points is expected to be limited. Conversely, when nanoparticles are administered at later time points (i.e., ∼4 h post-MPN), the vasodilation effect is likely to have subsided, and the observed effects would primarily reflect altered systemic distribution due to phagocytic modulation.

Toxicology analyses further indicate that both single and repeated MPN dosing regimens did not induce any observable short-term toxicity effects under the tested conditions. NO-mediated vasodilation has been previously explored as a strategy to enhance tumor perfusion and drug delivery, including using small-molecule NO donors and NO-releasing nanomaterials. While these approaches have shown the ability to transiently increase vascular permeability and improve tissue penetration, they are often associated with limitations such as rapid release kinetics, limited stability, and potential off-target effects due to systemic NO exposure. In contrast, the strategy presented here does not rely on the exogenous delivery of NO, but rather on the stimulation of endogenous NO production following MPN internalization by immune and, possibly, other cells. This mechanism may provide a more localized effect in malignant tissue, as seen in the CT26 tumor model, although this requires further validation with additional tumor models, and temporally regulated vasodilatory response, as it depends on nanoparticle-cell interactions and cellular activation rather than passive NO diffusion. In addition, the use of biocompatible components, such as albumin and methyl palmitate, may offer advantages in terms of safety and clinical translational potential. Nevertheless, we acknowledge that a direct comparison with established NO donors or other vascular-modulating delivery systems was not performed in this study, and further work will be required to systematically evaluate the relative benefits and limitations of these approaches.

## Conclusions

Our results demonstrate that MPN administration induces a rapid systemic vasodilation that appeared more pronounced in CT26 tumor vasculature than in healthy skin vasculature, as assessed by RSOM imaging analysis. Mechanistically, this early vasodilatory response, potentially mediated by NO release from macrophages and endothelial cells, likely enhances both vascular perfusion and vascular permeability and supports increased intratumoral uptake of macromolecules, such as 70 kDa dextran, and nanodrugs, such as the ∼30 nm Feraheme nanoparticles. Toxicology analyses further indicate that both single and repeated MPN dosing regimens are well tolerated in healthy mice.

Taken together with our previous findings, these data further suggest that the timing of MPN pretreatment can be strategically leveraged to optimize the delivery of therapeutic agents to tumors. Nanoparticles administered approximately 4 h after MPN injection primarily benefit from reduced liver uptake, whereas those administered within ∼15 min of pretreatment benefit from enhanced vascular permeability and extravasation into the tumor milieu. Because larger nanoparticles (>100 nm) are more susceptible to clearance and less capable of passive extravasation, they may be better suited to the longer pretreatment window, while smaller nanoparticles (15–30 nm) and macromolecules (∼70 kDa) are ideally positioned to exploit the early vasodilation phase.

Finally, these complementary mechanisms may be combined. Sequential MPN priming or staggered delivery of therapeutics could enable simultaneous exploitation of both perfusion-driven enhancement and macrophage suppression, offering a flexible strategy to improve the delivery and efficacy of macromolecules and nanomedicines across a range of sizes and therapeutic modalities.

## Materials and Methods

### Fabrication and Characterization of Methyl Palmitate Nanoparticles

Methyl palmitate nanoparticles (MPN) were produced by using a self-assembly method: a solution 1:1 (V:V) of methyl palmitate (Merck—Sigma-Aldrich—Germany) and ethanol containing 2 mg of methyl palmitate was added to 100 μL of a 50 mg/mL BSA (Merk—Sigma-Aldrich—Germany) solution. The obtained mixture was sonicated for 1 min into a water bath, washed in 1 mL of PBS (Thermo Fisher Scientific, USA) and centrifuged at 15,000 rpm at 4 °C. Particles were washed and finally resuspended in 1 mL of PBS or water (depending on the specific need) and sonicated for 1 min. Average size, size distribution, and zeta potential of MPN were analyzed using dynamic light scattering. Samples were diluted with isosmotic double distilled water (1:100 v/v) to avoid multiscattering phenomena and analyzed at 25 °C with a Zetasizer Nano (Malvern, U.K.), equipped with a 4.5 mW laser diode and operating at 670 nm as a light source, and the scattered photons were detected at 173 °C. A third order cumulative fitting autocorrelation function is applied to measure the average size and size distributions. The analysis was carried out according to the following instrumental setup: (a) a real refractive index of 1.59; (b) an imaginary refractive index of 0.0; (c) a medium refractive index of 1.330; (d) a medium viscosity of 1.0 mPa·s; and (e) a medium dielectric constant of 80.4. Transmission electron microscopy (TEM) micrographs were acquired using JEOL JEM 1011 (Jeol, Japan) electron microscope (Electron Microscopy Facility, Fondazione Istituto Italiano di Tecnologia, Genoa—Italy) operating with an acceleration voltage of 100 kV and recorded with a 11 Mp fiber optical charge-coupled device (CCD) camera (Gatan Orius SC-1000). MPN samples were diluted 1:100, dropped on 150-mesh glow discharged “Ultrathin” carbon-coated Copper TEM grids, dried and directly observed.

### Cell Cultures

RAW264.7 cells were purchased from the American Type Culture Collection (ATCC, Rockville, MD, USA) and maintained in Dulbecco’s Modified Eagle’s Medium high-glucose (DMEM) (Euroclone—Italy) supplemented with 10% fetal bovine serum (ATCC) and 1% penicillin/streptomycin. Cells were grown at 37 °C in an 80% humid atmosphere of 5% CO_2_. Bone Marrow Derived Monocytes (BMDMs) from rats and from mice were isolated based on the following procedures. Briefly after specifying the animal, femurs were isolated, cleaned from surrounding tissues and washed in PBS (Thermo Fisher Scientific, USA), a cut was performed at both ends. PBS was used to flush the cavities, cells were harvested and plated in media supplemented with macrophage Colony-Stimulating Factor (mCSF) (10 ng mL^–1^) (Merk—Sigma-Aldrich—Germany). The procedures were conducted following the guidelines of the Institutional Animal Care and Use Committee of IIT and the National Institutes of Health guide for the care and use of Laboratory animals (NIH Publications No. 8023, revised 1978). Culturing medium was changed after 3 days to remove unattached cells. BMDMs were used on the following day. Culture media: DMEM supplemented with 15% FBS, 1% penicillin/streptomycin, and rat M-CSF (Merk—Sigma-Aldrich—Germany) (according to vendor indications).

Human Umbilical Vein Endothelial Cells (HUVEC) were cultured using Vascular Cell Basal Medium supplemented with 0.2% bovine brain extract, rhEGF (5 ng/mL), l-glutamine (10 mM), heparin sulfate (0.75 units/mL), hydrocortisone (1 μg/mL), ascorbic acid (50 μg/mL), 2% fetal bovine serum, and 1% penicillin/streptomycin. CT26 cells were cultured in RPMI base media supplemented with 10% fetal bovine serum and 1% penicillin/streptomycin. Cells were cultured under controlled environmental conditions (37 °C in 5% CO2).

### Particle Internalization Analysis via Confocal Microscopy

For cells imaging 65,000 μ_B_DM, 65,000 RAW 267.4, and 6,500 HUVEC, were separately seeded into each well of a μ-Slide 8 well high (ibidi GmbH—Germany) maintaining culturing conditions, as described above. Cells were treated overnight with Cy5-MPN. After these treatments media was removed and cells were washed in PBS (Thermo Fisher Scientific, USA). Fixation was performed using a 3.7% solution of paraformaldehyde (Sigma-Aldrich, USA) for 10 min; 3 washes with PBS (Thermo Fisher Scientific, USA) were performed after cell fixation. To highlight cell body BMDM were immunostained for alpha tubulin, while RAW 267.4 and HUVEC were stained for actin. For BMDM α-Tubulin antibody (Sigma—T5168) was used as primary antibody and Abcam—ab6786 was used as secondary antibody according to vendors indications. Actin was stained using Alexa Fluor 488 Phalloidin (Thermo Fisher Scientific, USA) according to vendor indication. For all the analyses, nuclei were stained using DAPI (Thermo Fisher Scientific, USA) following vendor indications. A 63× objective was used and a z-stack series was acquired (≥12 steps of 1,000 nm each were acquired per image).

### Transmission Electron Microscopy Analysis of BMDM

Transmission electron microscopy (TEM) micrographs were acquired using JEOL JEM 1011 (Jeol, Japan) electron microscope (Electron Microscopy Facility, Fondazione Istituto Italiano di Tecnologia, Genoa—Italy) operating with an acceleration voltage of 100 kV and recorded with a 11 Mp fiber optical charge-coupled device (CCD) camera (Gatan Orius SC-1000, USA). 500000 μ_B_DMs were seeded into each well of a 6 wells plate. A sterilized borosilicate glass was previously placed into each well; culturing conditions were maintained as described above. Cells were treated overnight with MPN, samples were fixed for 2 h in 1.5% glutaraldehyde in 0.1 M sodium cacodylate buffer (pH 7.4), post fixed in 1% osmium tetroxide in the same buffer and stained overnight with 1% uranyl acetate aqueous solution. Samples were then dehydrated in a graded ethanol series, infiltrated with a series of ethanol/resin solutions and finally embedded in epoxy resin (Epon 812, TAAB). Thin sections were cut with a Leica UC6 ultramicrotome (Leica Microsystems, Germany) equipped with a diamond knife (Diatome).

### Griess Assay

100,000 μ_B_DM and 10,000 HUVEC were seeded in 24 well plates (Corning—USA) in 500 μL of media respecting culturing conditions above indicated. Cells were treated varying the MPN concentration from 0 to 0.25 mM equivalent dose of methyl palmitate. After the different time of treatment the media was collected, centrifuged (500 RCF, 5 min, 4 °C) and analyzed.

### Animal Studies Ethics Statement

All animal experiments were performed following the protocols evaluated and approved by the Institutional Animal Care and Use Committee (IACUC) of Memorial Sloan Kettering Cancer Center (Ethics Approval Number #08-07-014).

### Raster-Scanning Optoacoustic Mesoscopy (RSOM)

Nude mice at 6–8 weeks of age were obtained from Jackson. The tumor-bearing cohort were implanted subcutaneously, between the flank and the dorsal side of the mouse, with 2 million CT26 cells resuspended in 100 μL of PBS. Once tumors reached 500–1000 mm^3^, mice were randomized into PBS or MPN injection groups. For RSOM imaging, mice were placed in an isoflurane chamber for 5 min before being positioned on the RSOM imaging bed with continuous isoflurane and oxygen flow. Ultrasound gel was applied to the mouse in the region designated for RSOM imaging. The RSOM imaging head containing the laser, waterbed, and transducer (iThera) were placed on the dorsal side of the mouse containing tumors or healthy skin (no tumor). Once an appropriate area and depth was set with the imaging head, images were then acquired before MPN or PBS injection. Mice were then injected with either 300 μL of PBS or MPN (resuspended in PBS) and image acquisition was initiated at 1 min post injection (T1). Additional images were acquired every 5 min post T1 until the 20 min time point.

The RSOM Viewer program was used to complete motion correction and spectral unmixing on the RSOM images. Spectral unmixing removes noise from melanin and air bubbles and allows for the imaging of oxygenated and deoxygenated blood, as well as high and low frequency wavelengths, corresponding to narrow or constricted vessels or wider/dilated vessels, respectively.

For vessel width analysis, 21–25 vessel regions were chosen at random on blinded images of PBS or MPN injected mice, with the only condition being that there was little background noise surrounding the region of the vessel being analyzed. ImageJ was used to create a stack of the T1 and T4 images, and the plot profiler tool was used to plot the gray value across the length of a 12-pixel line drawn over the vessel/region of interest. The maximal gray value is then located at the center of the 12-pixel line and is used to normalize all other gray values, and generate a bell curve representing normalized gray value against pixel length, with the largest gray value equaling 1, once normalized. A python script was used to interpolate the pixel values (x1 and x2) at full width half-maximum (y = 0.5) for T4 and T1. The change in vessel width (pixel change) from T4 to T1 was then determined by calculating (T4 × 2 – T4 × 1) – (T1 × 2 – T1 × 1), referred to here as ΔΔX. The python script used to calculate the ΔΔX between T1 and T4 time points for all the vessel regions analyzed, can be found at: https://github.com/liz-lemon18/Delta_Delta_X/blob/main/delta_delta_x.py. The pixel-to-micron conversion for vessel width measurements was based on specifications provided by iThera for the RSOM P50 Explorer, where 1 pixel corresponds to 20 μm.

Vessels which appeared anew in the T4 time point but were not visible in the T1 time point could not be analyzed, since a plot profile could not be generated for nonvisible vessels (nondetectable pixels) in T1. Therefore, this analysis focuses mainly on vessels whose change in width could be measured by the ImageJ plot profile function in T4 vs T1.

### ^89^Zirconium-Labeled Feraheme Biodistribution Analysis

6–8 weeks old BALB/c mice obtained from Taconic were implanted in the flank region with 2 million CT26 cells that were suspended in 100 μL of phosphate buffered saline. Once tumors reached a volume between 250–1,000 mm^3^, mice were randomized and placed into PBS or MPN preinjection groups. For injections, mice were placed into a cage for heating for 5 min, after which mice were injected with 300uL of PBS or MPN via tail vein injection and then placed in a second cage for a 5 min recovery period. Mice were then anesthetized by using isoflurane for 5 min, immediately after which 100 μL of ^89^Zr-FH (at a concentration of ∼1.2 mg of iron and ∼130 μCurie of ^89^Zr per dose) was injected retro-orbitally. Six hours post injection, mice were imaged using the Siemens Inveon PET/CT imager. Image processing was completed using the Inveon Research Workplace software to quantify the PET signal from sagittal slices of 3D maximum intensity projections (MIPs) of the CT26 tumor mass in PBS and MPN preinjected mice, using the paintbrush tool to highlight the region of interest (ROI). Mice with tumors of equivalent masses, resulting in near identical average tumor mass and variability among the two pretreatment groups, were compared for this experiment (n = 4). The mean intensity of the ROIs was then divided by the volume of the tissue analyzed in the 3D MIP to obtain volume-normalized mean intensities for each group.

For gamma count analysis, mice were sacrificed at 24 h post ^89^Zr-FH injection and organs were collected into preweighed 5 mL plastic capped tubes. Each sample was read for 60 s on the gamma counter to obtain counts per minute (CPM) and an ^89^Zr standard curve was used to convert CPM to activity in microcuries (μCi). After decay correction calculations were completed, data was reported as percent injected dose per gram of tissue (%ID/g) after weighing 5 mL plastic capped tubes once again, post organ harvesting. Percent change was calculated using the percent change equation (shown below) in which the averaged PBS (*Average*_PBS_) 6-h region-of-interest (ROI) or 24 h %ID/g value for the CT26 tumor mass was subtracted from *each* MPN (*Value*_MPN_) ROI or %ID/g value. Percent change of PBS injected mice was calculated in a similar manner (equation shown below), in order to display the spread of data points around the mean.

$$\mathrm{Percent change MPN}=\frac{Value_{MPN}-Average_{PBS}}{Average_{PBS}}\times 100$$

$$\mathrm{Percent change PBS}=\frac{Value_{PBS}-Average_{PBS}}{Average_{PBS}}\times 100$$

### Intratumoral Dextran Accumulation

BALB/C mice of 6–8 weeks of age were obtained from Taconic and were subcutaneously implanted in the flank with 2 × 10^6^ CT26 cells in 100 μL of PBS. Once tumors reached ∼400–2,000 mm^3^, mice were randomized in MPN or PBS preinjection groups (5 mice per group). One mouse was set aside for PBS only or MPN only negative control injections. 300 μL of PBS or MPN was injected via tail vein, after which, mice were anesthetized with isoflurane and 100 μL of Dextran-Oregon Green (0.83 mg/mouse) was injected retro-orbitally, within 15–20 min of MPN or PBS injection. 24 h later, mice were sacrificed and tumors were collected and fixed in 4% paraformaldehyde for 1 week at 4 °C. Tumor tissues were then washed twice with PBS without calcium and magnesium and incubated in a 40% sucrose and PBS solution for 5 days. Tissues were then embedded in OCT and cut into 10 μm slices and mounted onto microscope slides. Slides were imaged and scanned using a 488 nm laser. Tumor tissues harvested from mice that received the PBS or MPN preinjection but no Dextran-Oregon Green injection (negative control) were used as negative control and for background subtraction. Slice analysis was conducted by calibrating 2 thresholds on the green channel, one permissive for measuring the slide area and one more restrictive to indicate the signal coming from the dextran. The positive area fraction (%) was calculated by dividing the area occupied by the dextran signal and the total area of the slide. For this experiment *n* ≥ 8 animals per group were considered with *n* > 12 slide replicates per animal. Radial profile and concentric areas analysis were run by using ImageJ (n = 8 animals per group); ANOVA single factor was used for P-value calculation.

### Blood Tests

Healthy immunocompetent C57BL/6 mice were treated with MPN once with a high dose of methyl palmitate = 3.75 mg/20 g, or twice per week (for 2 weeks) with a lower dose of methyl palmitate = 0.94 mg/20 g. The injections were performed in the tail vein. 24 h after the last treatment, the blood was withdrawn by the retro-orbital plexus by using a heparinized glass capillary (Sigma-Aldrich, USA). For the hematology tests the blood of each animal was transferred into a Microvette CB 300 EDTA K2E (Sarstedt, Germany). For the biochemistry analyzes the blood of each animal was transferred into an Eppendorf tube 1.5 mL (Eppendorf, Germany) and centrifuged for 10 min at 10,000 rpm at room temperature. Serum was isolated and stored at −80 °C before proceeding to the analyses. The tests were run at the “Mouse Clinic” of Ospedale San Raffaele in Milan.

### Histology Analyses

Healthy immunocompetent C57BL/6 mice were treated with MPN once with a high dose of methyl palmitate = 3.75 mg/20 g, or twice per week (for 2 weeks) with a high and a low dose of methyl palmitate: 3.75 mg/20 g and 0.94 mg/20 g, respectively. The injections were performed in the tail vein. 24 h after the last treatment mice were sacrificed by using CO_2_, the same animal used for blood tests were used for histology (plus the additional group missing in the other analysis). Main organs were collected and washed in PBS. For the liver, the lobe 2 was used for the analyses. Kidneys were split in half following the longer axis. For the lungs, the left lung was used for the analyzes. Heart was transversally split in two halves having 1 atrium and 1 ventricle per part. Spleen was maintained integer, and no cut was performed. All the samples from one animal were isolated into a single glass vial after an additional PBS wash. Glass vials were kept in ice and PFA 4% was added in order to fully cover all the sample. Vials were kept at 4 °C for 48 h, PFA 4% was removed and 3 PBS washes were performed. After the third wash the PBS was completely removed and ethanol 70% was added, allowing all the samples to be immersed. Each organ was then disposed into a single bio cassette for embedding. All the cassettes were collected into 500 mL jars and shipped at room temperature to the “Mouse Clinic” of Ospedale San Raffaele in Milan and processed there.

## Supplementary Material



## Notes

SI1: The DLS analysis of Cy5-MPNs, which were used in confocal microscopy experiments for the analysis of MPN internalization in BMDM, RAW 264.7, and HUVEC; SI2: The monitoring of calcium signaling in response to MPN treatment in BMDM, showing individual cells exhibiting different rates of Ca^2+^ increase; SI3: Additional Raster Scanning Optoacoustic Mesoscopy (RSOM) of mouse healthy (nontumor) tissue after injection with PBS or MPN; SI4: Additional Raster Scanning Optoacoustic Mesoscopy (RSOM) of CT26 mouse tumor tissue after injection with PBS or MPN; SI5: Additional histology slices of CT26 tumors showing dextran distribution in the presence or absence of MPN pretreatment; SI6: Example of concentric areas subdivision of a CT26 tumor slice (a); additional plot of dextran fluorescence intensity in different concentric areas of CT26 tumor slices from mice that were pretreated or not with MPN (b); SI7: PET/CT images of ^89^Zr-FH-injected mice preinjected with PBS or MPN, acquired 6 h post injection with ^89^Zr-FH, after (a) PBS preinjection or (b) MPN preinjection; SI8–11: Serological and hematological blood parameters in C57BL/6 mice that received a single high dose or multiple lower doses of MPN systemically (PDF)
