# Supplementary material for: Enhancing Tumor Perfusion and Nanomedicine Delivery by Modulating Vascular Tone with Methyl Palmitate Nanoparticles
Source: ACS Nano. 2026 Jul 17;20(30):21172–88. doi: 10.1021/acsnano.6c03988 (PMC13456299; doi:10.1021/acsnano.6c03988)
Supplement: Supplementary file 1 [file nn6c03988_si_001.pdf]

SUPPORTING INFORMATION:

ENHANCING TUMOR PERFUSION AND NANOMEDICINE DELIVERY BY MODULATING VASCULAR TONE WITH METHYL PALMITATE NANOPARTICLES

Roberto Palomba[1 ♦], Elizabeth Isaac[2 ♦], Raffaele Spanò[1], Federica Piccardi[1], Benedict McLarney[2], Elana Apfelbaum[2], Nermin Mostafa[2], Hsiao-Ting Hsu[2], Jan Grimm[2 ♣] and Paolo Decuzzi [1,3 ♣]

[1] Laboratory of Nanotechnology for Precision Medicine – Fondazione Istituto Italiano di Tecnologia, Via Morego 30, 16163, Genova, Italy

[2] Memorial Sloan Kettering Cancer Center, Sloan Kettering Institute (SKI), Molecular Pharmacology Program, 1275 York Avenue, New York City, NY Z2025 - USA

[3] School of Medicine/Division of Oncology, Center for Clinical Sciences Research, Stanford University, 269 Campus Drive, Stanford, CA 94305 - USA

♦ Shared first authorship

♣ corresponding authors

Corresponding authors: Jan Grimm (grimmj@mskcc.org) and Paolo Decuzzi (paolo.decuzzi@iit.it)

Supporting Information 1:

Dynamic light scattering (DLS) analysis of Cy5-MPN.

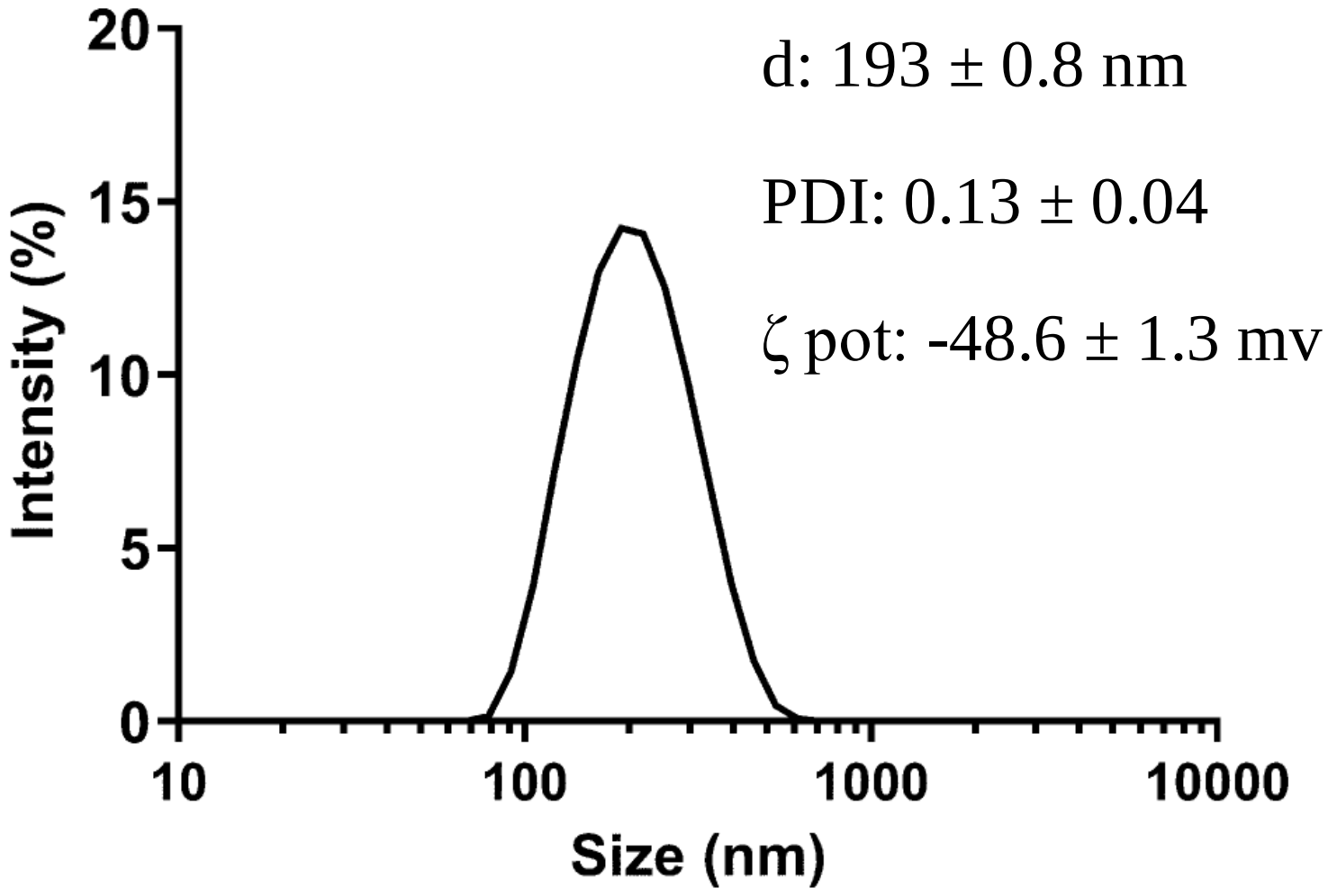

Dynamic light scattering (DLS) analysis showing the hydrodynamic size distribution of Cy5-MPN, including diameter (d), polydispersity index (PDI), and surface electrostatic potential (ζ).

Supporting Information 2:

Calcium signaling monitoring in response to MPN treatment in BMDM.

we used time-lapse fluorescence microscopy to monitor intracellular Ca^{2+} levels using the calcium-sensitive dye Fluo-4 AM. BMDM were seeded in microscopy chamber slides, loaded with Fluo-4 AM, and exposed to MPN immediately before image acquisition. Selected frames from the time-lapse recordings are shown for three different representative cells. The fluorescent intensity measured in the region of interest (ROI) associated with the cells increases with time documenting the progressive rise in intracellular Ca2+ concentrations (**SI2a**). During the first few minutes, the intracellular Ca2+ levels steadily increased to reach a maximum around 10 min (**SI2b**). Notably, individual cells exhibited different rates of Ca^{2+} increase, but all tended to reach comparable levels within the first 10 minutes.

a

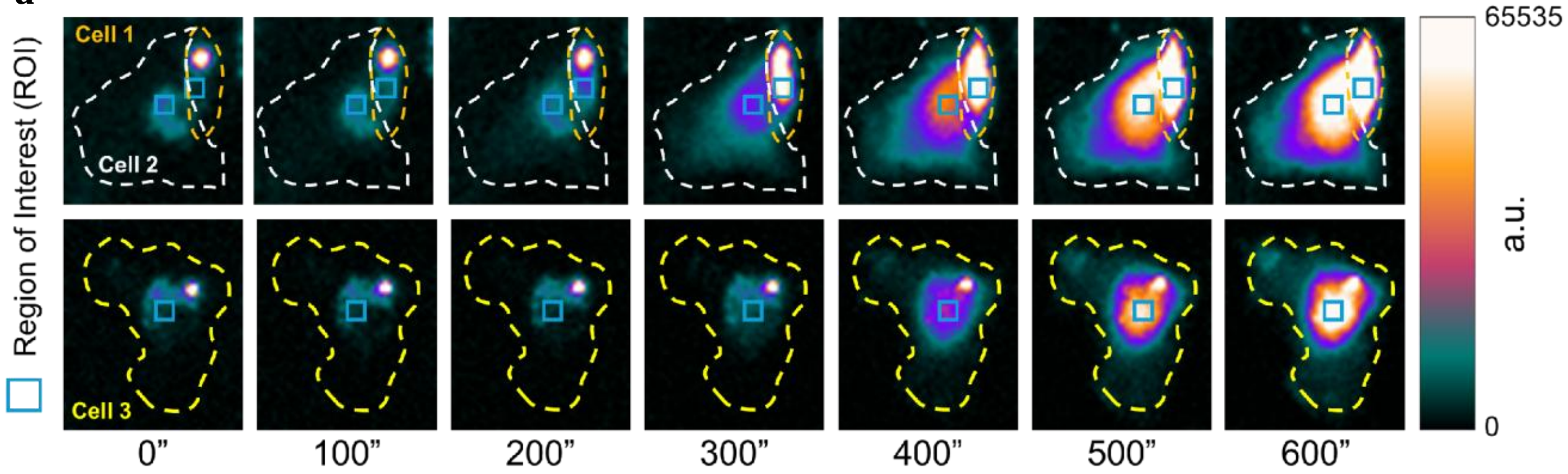

b

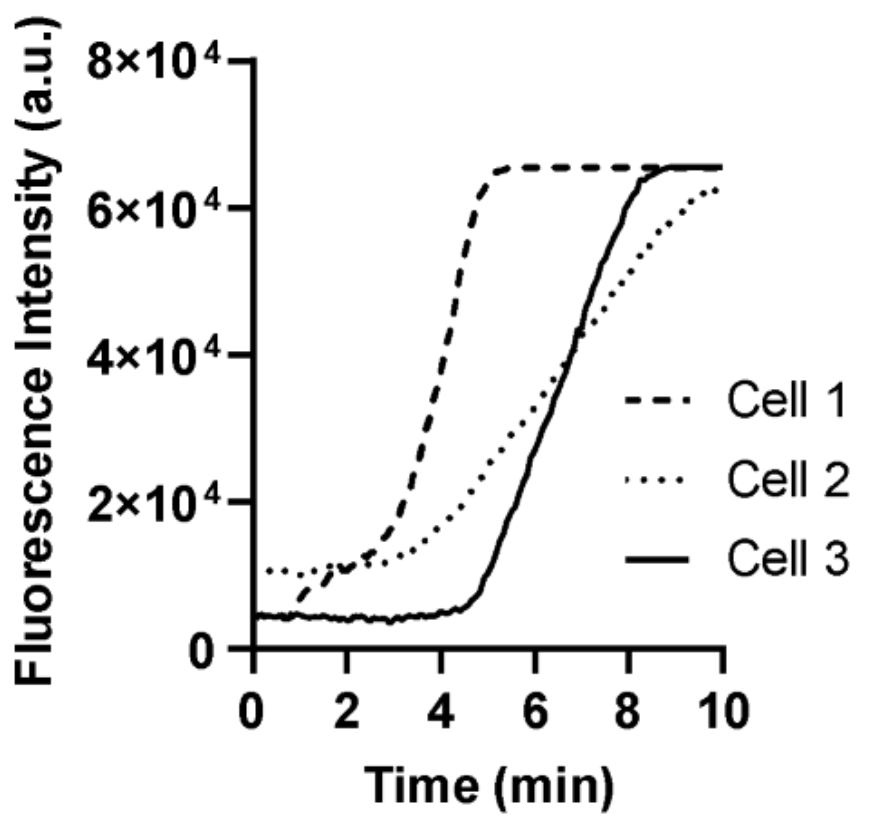

Single-cell Ca^{2+} responses observed by time-lapse microscopy following MPN exposure. (white and yellow dashed lines identify the entire cell body; azure boxes identify the region of interest (ROI) for Ca^{2+} level measurements as in 2b (**a**). Time course of intracellular Ca^{2+} concentration in BMDM measured by live-cell fluorescence imaging immediately after exposure to MPN (**b**).

Methods: 65.000 BMDMs were seeded into each well of a µ-Slide 8 Well high (ibidi GmbH – Germany) maintaining culturing conditions, as described above. For monitoring intracellular calcium concentration Fluo-4AM, cell permeant (Thermo Fisher – USA) was added to the media according to vendor indication. 1 day after the seeding cells were treated with MPN. For this treatment 1 batch 1× of MPN was dispersed in PBS and sonicated right before the use, 7.5 µL of the suspension was added to the media. Cells were imaged right after by time lapse microscopy using a Nikon Eclipse-Ti-E microscope (Nikon Instrument Inc. – Japan) under controlled environmental conditions. Movies were acquired at a frame rate of 12 fpm using a 40× lens. The events registered from 3 cells were acquired.

Supporting Information 3:

Raster Scanning Optoacoustic Mesoscopy of Healthy Tissue.

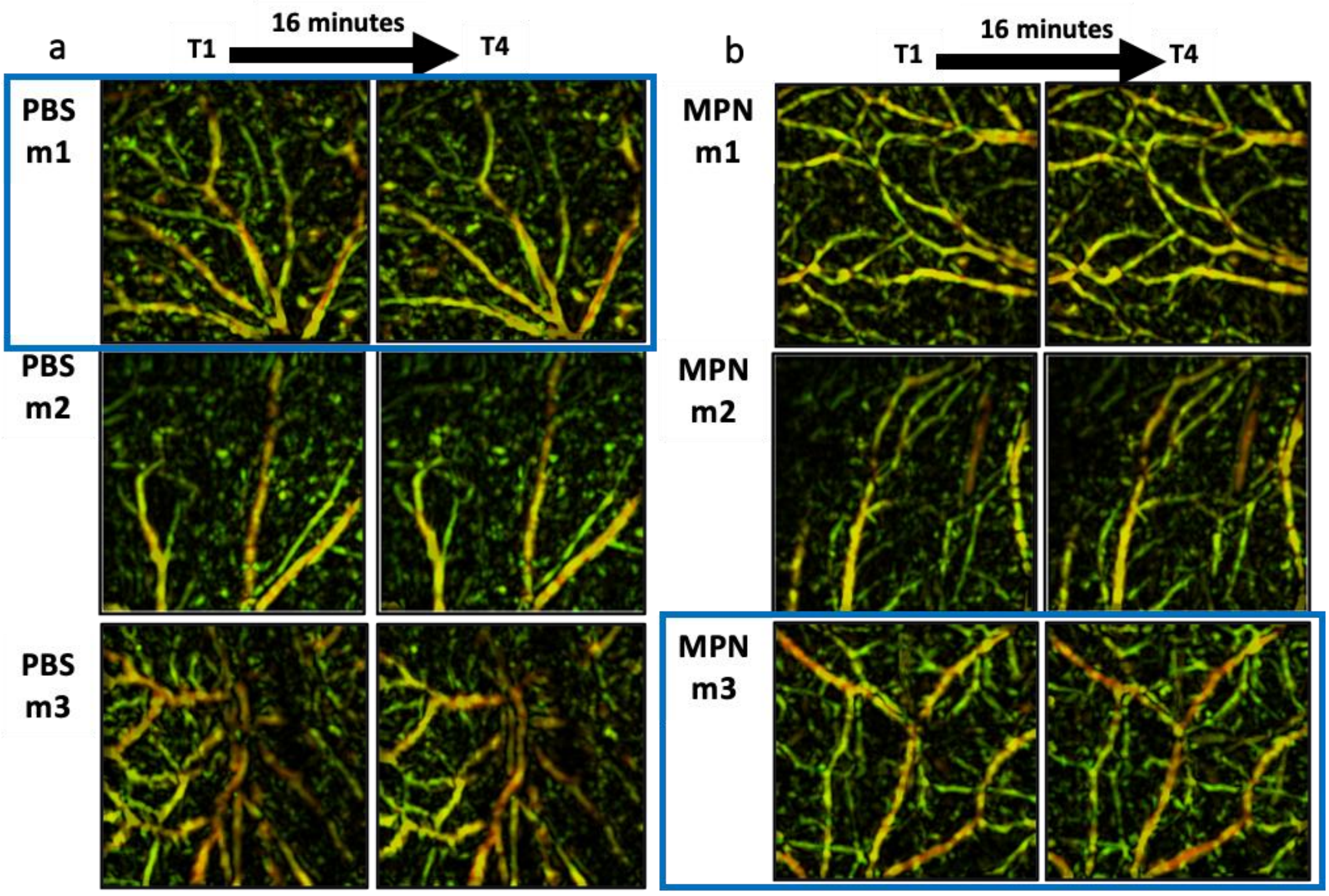

Raster Scanning Optoacoustic Mesoscopy (RSOM) of mouse healthy (non-tumor) tissue after injection with PBS or MPN at timepoints T1 (1 minute post injection) or T16 (16 minutes post injection) in n=3 mice. Representative images chosen for use in the main text are outlined with blue boxes.

Supporting Information 4:

Raster Scanning Optoacoustic Mesoscopy of Tumor Tissue.

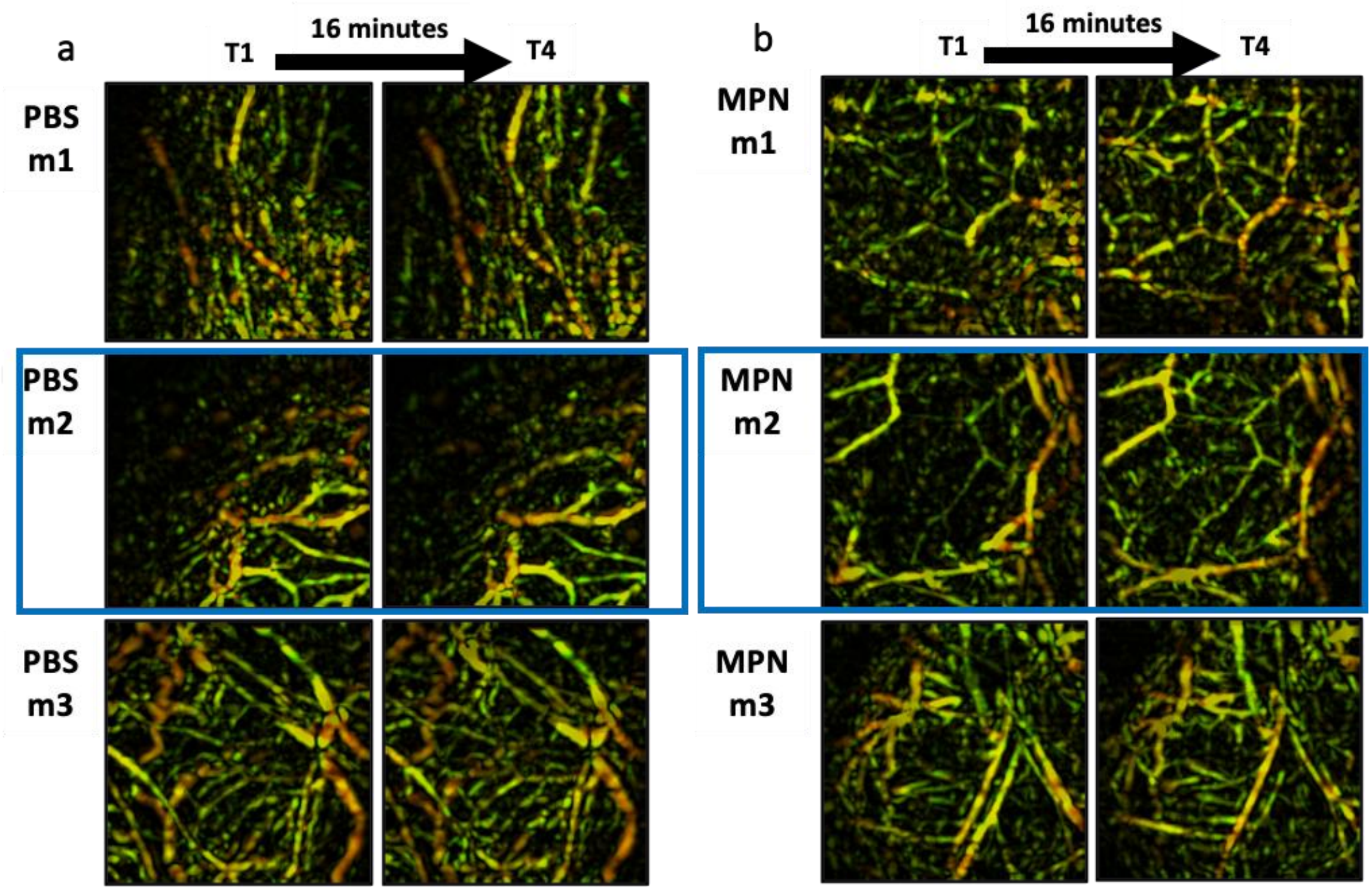

Raster Scanning Optoacoustic Mesoscopy (RSOM) of mouse CT26 tumor tissue after injection with PBS or MPN at timepoints T1 (1 minute post injection) or T16 (16 minutes post injection) in n=3 mice. Representative images chosen for use in the main text are outlined with blue boxes.

Supporting Information 5:

Dextran Distribution in Tumor Tissue (Additional Images).

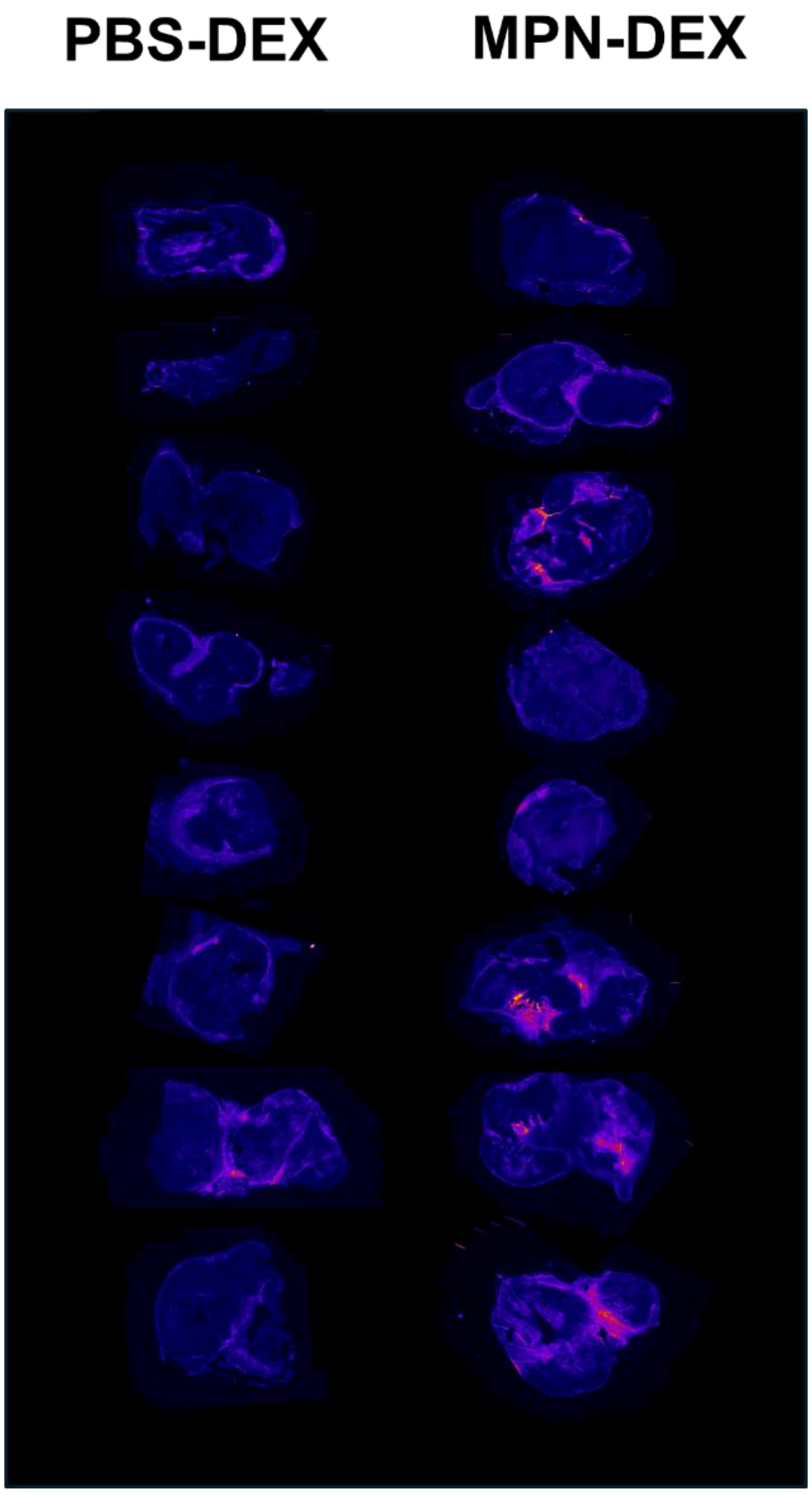

Randomly picked tumor slices in PBS-injected and MPN-pretreated mice.

Supporting Information 6:

Dextran Fluorescence intensity calculated in concentric areas of the tumor.

a

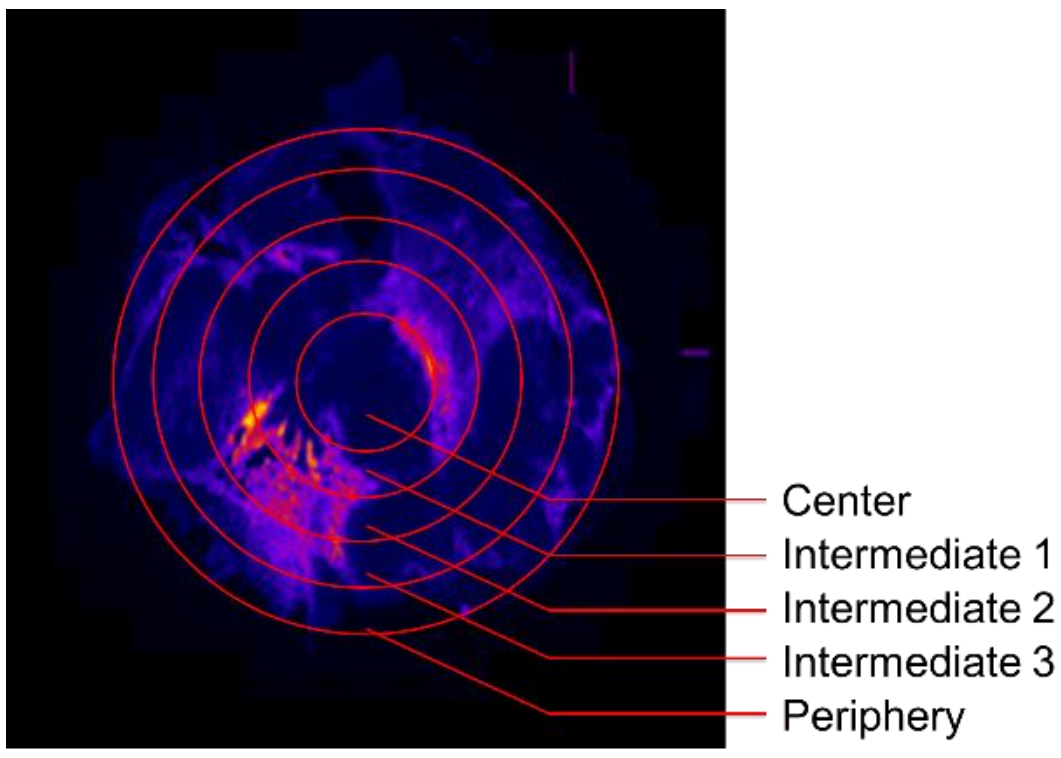

b

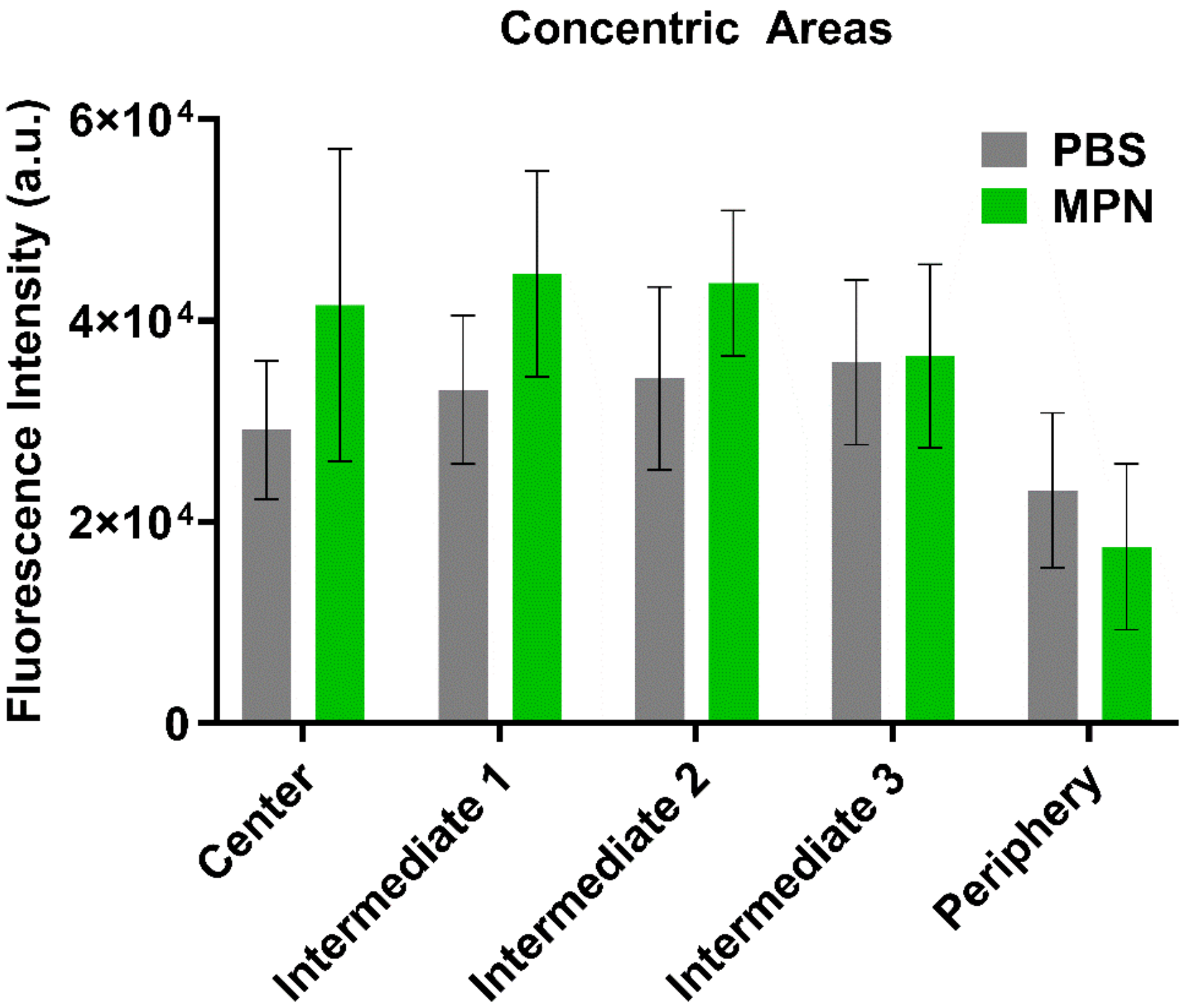

Dextran fluorescence intensity calculated in concentric ROIs of tumor slices. a, example of the ROIs distribution scheme. b, fluorescence intensity plot. P-values (PBS vs MPN) by following the order of columns: 0.038; 0.024; 0.035; 0,945; 0,157.

Supporting Information 7:

PET/CT images of ^{89}Zr-FH injected mice pre-injected with PBS (a) or MPN (b).

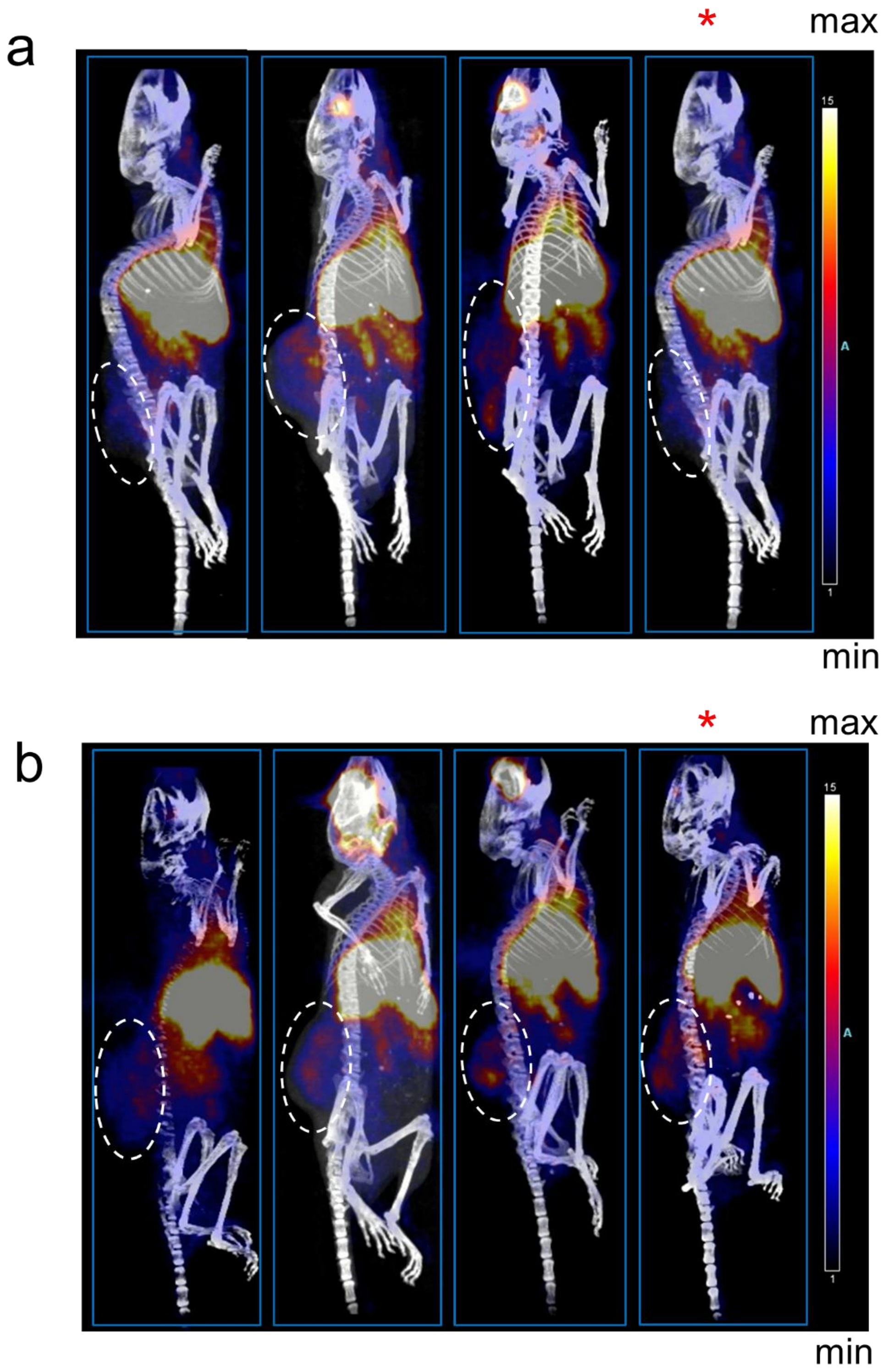

PET/CT images of n=4 mice acquired 6-hours post injection with ^{89}Zr-FH. Mice chosen as representative images are marked with an asterisk (*) symbol. Tumors are indicated with dashed circles.

Supporting Information 8:

Serological Blood Parameters in mice that received a single high dose of MPN.

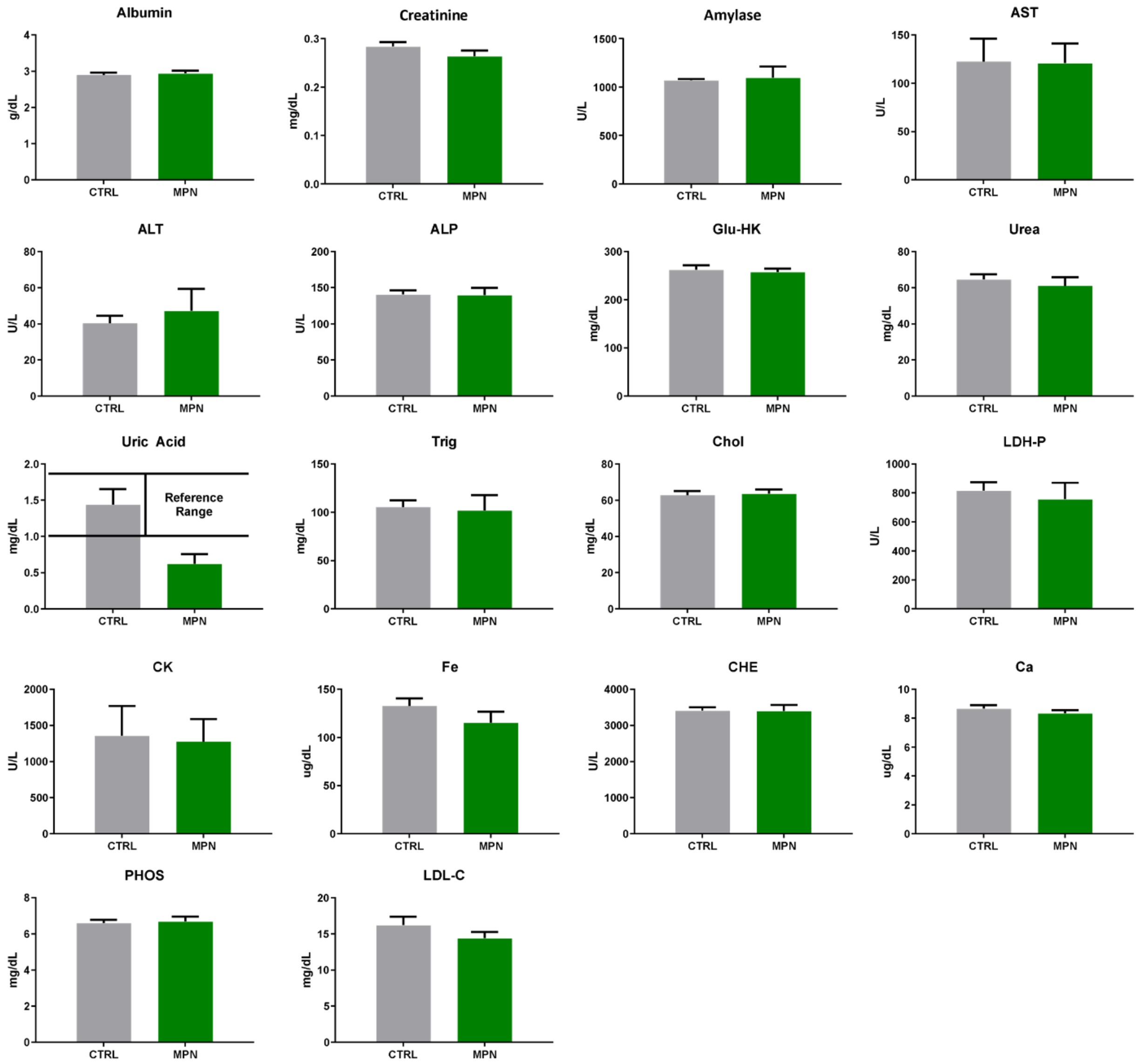

Serological blood parameters in C57BL/6 mice that received a single high dose of MPN corresponding to 3.75 mg of methyl palmitate per 20 g body weight, administered via tail-vein injection.

Supporting Information 9:

Hematological Blood Parameters in mice that received a single high dose of MPN.

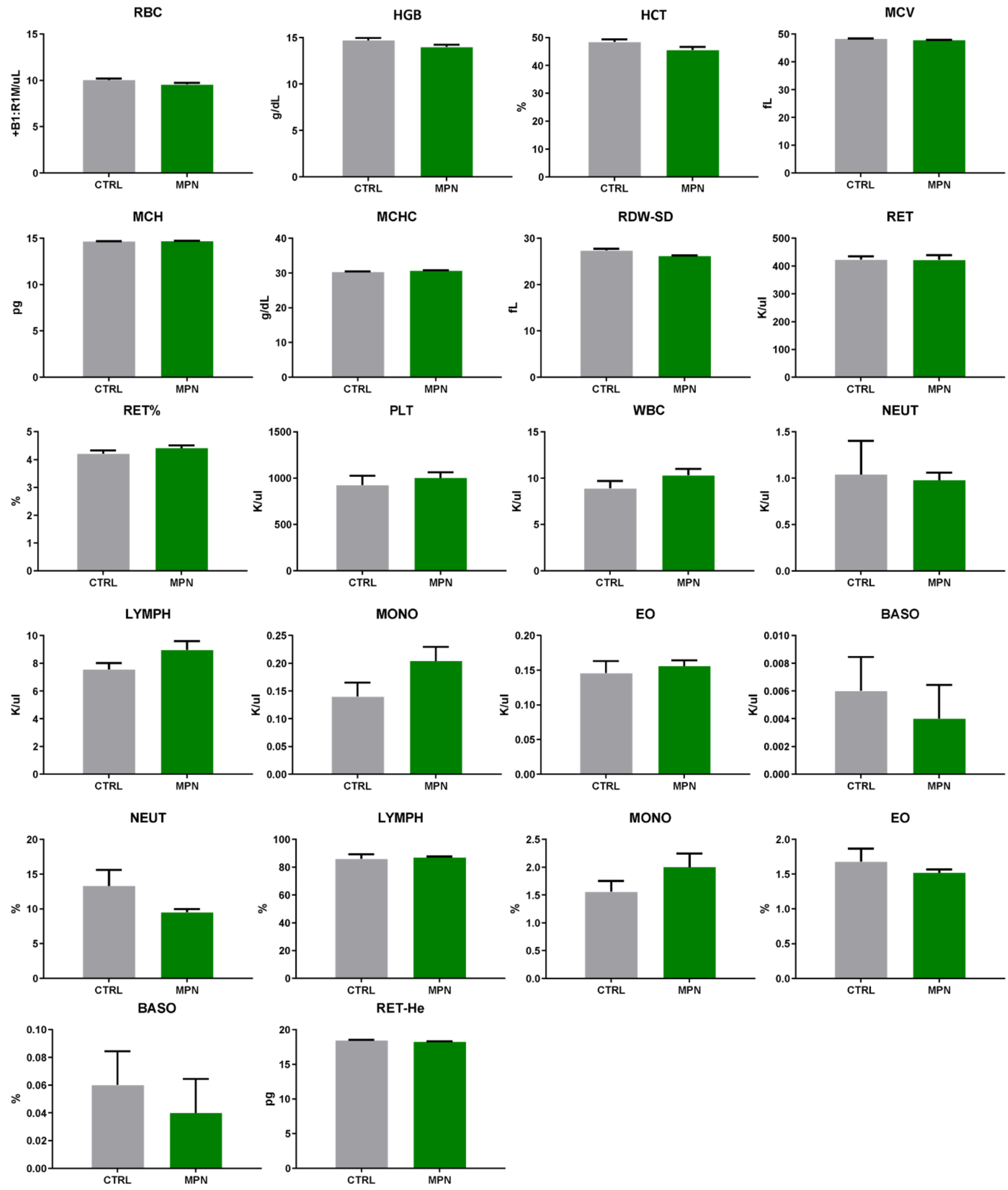

Hematological parameters in C57BL/6 mice that received a single high dose of MPN corresponding to 3.75 mg of methyl palmitate per 20 g body weight, administered via tail-vein injection

Supporting Information 10:

Serological Blood Parameters in mice received four injections of MPN at lower dose.

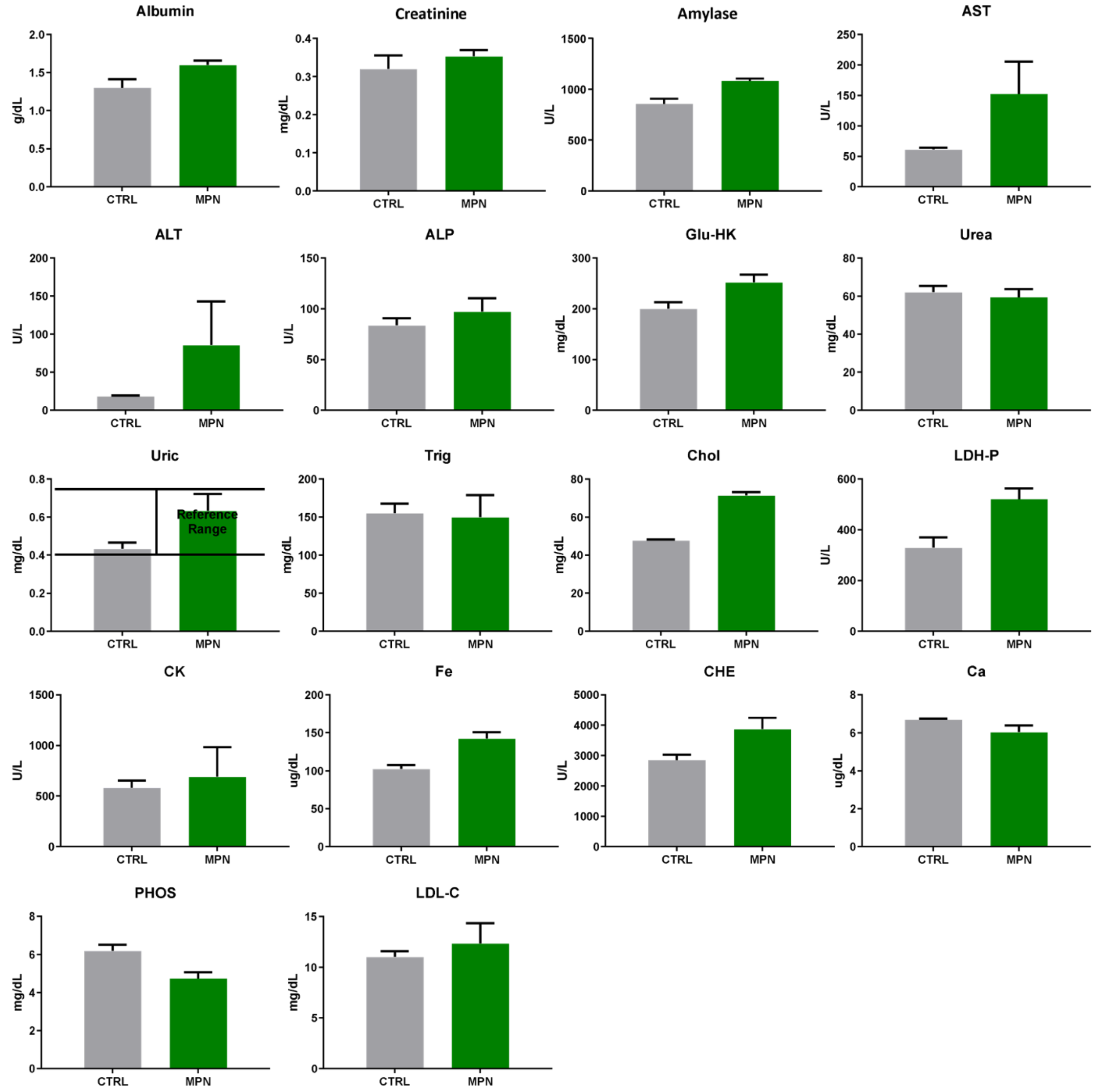

Serological blood parameters in C57BL/6 mice received four injections of MPN administered twice per week over a 2-week period at a lower dose equivalent to 0.94 mg of methyl palmitate per 20 g body weight, administered via tail-vein injection.

Supporting Information 11:

Hematological Blood Parameters in mice received four injections of MPN at lower dose.

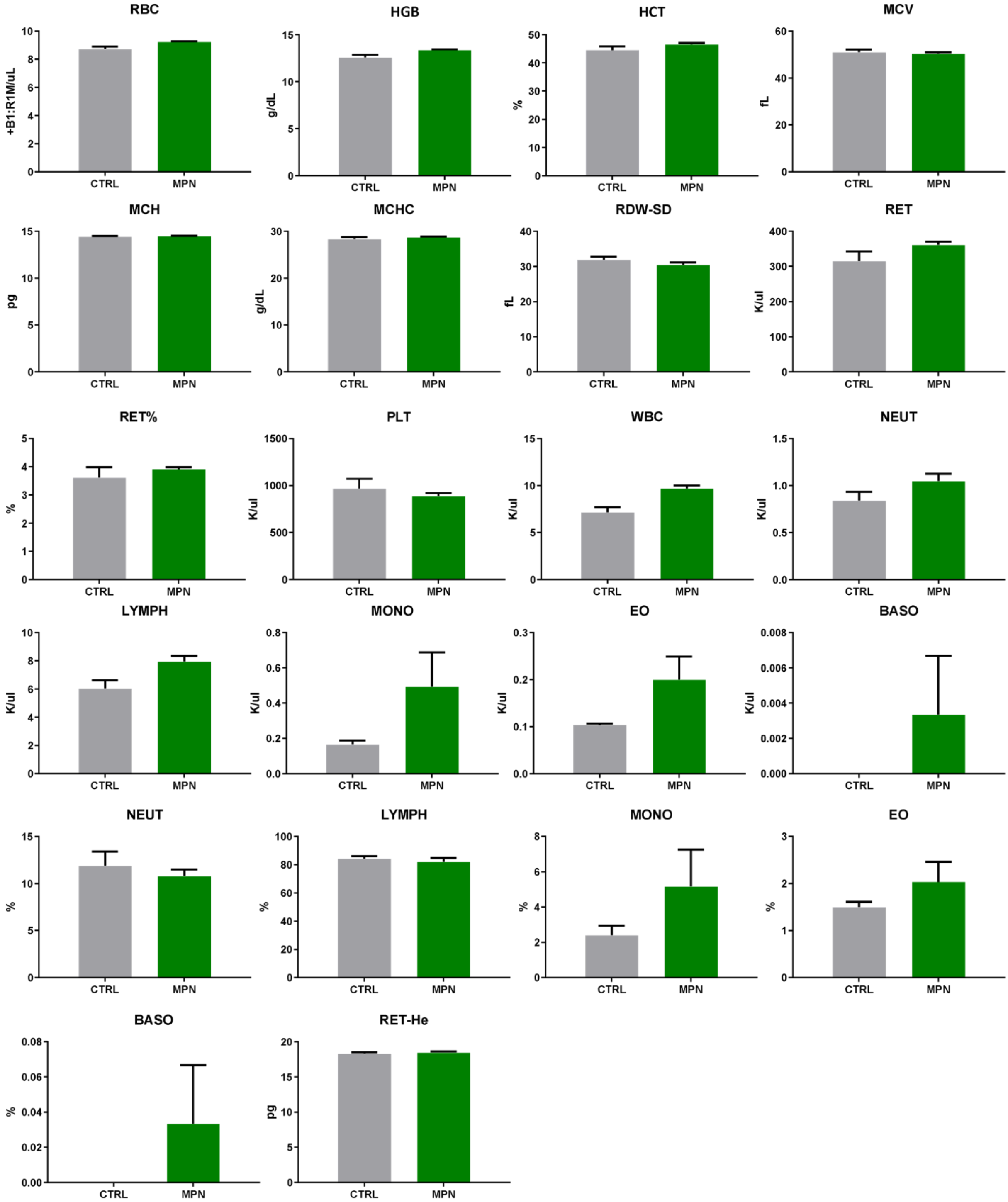

Hematological parameters in C57BL/6 mice received four injections of MPN administered twice per week over a 2-week period at a lower dose equivalent to 0.94 mg of methyl palmitate per 20 g body weight, administered via tail-vein injection.
